# Supplementary material for: A conserved archaeal protein with evolutionary links to bacterial ribosome hibernation and eukaryotic energy sensing
Source: EMBO J. 2026 Oct 2;45(21):7564–81. doi: 10.1038/s44318-026-00918-6 (PMC13728122; doi:10.1038/s44318-026-00918-6)
Supplement: Supplementary file 1 [file 44318_2026_918_MOESM1_ESM.pdf]

Appendix for "*A conserved archaeal protein with evolutionary links to bacterial ribosome hibernation and eukaryotic energy sensing*"

TABLE OF CONTENTS

Appendix Table S1 3
Appendix Table S2 4
Appendix Figure S1 5
Appendix Figure S2 6
Appendix Figure S3 7
Appendix Figure S4 8
Appendix Figure S5 8
Appendix Figure S6 9
Appendix Figure S7 9
Appendix Figure S8 10
Appendix Figure S9 11

APPENDIX TABLES

Appendix Table S1. CryoEM data collection, processing, model building, and validation information.

**Data acquisition**						
Microscope	Titan Krios G3i					
Camera	Gatan K3 (counting mode)					
Magnification (X)	81,000					
Voltage (kV)	300					
Total electron dose ($e^-/Å^2$)	50.51					
Defocus range (µm)	-0.2 to -2.4 (step size: 0.2)					
Pixel size (Å)	1.06					
Micrographs collected (#)	9,140					
**Map reconstruction**						
**Reconstruction**	**AHA + E/E-tRNA**	**AHA alone**	**P/P-tRNA**	**A/A-, P/P-tRNA**	**P/P- , E/E-tRNA**	**A/P-, P/E-tRNA**
Processing suite	cryoSPARC					
Symmetry	C1					
Final particle count	82,088	70,830	27,005	17,522	27,489	12,771
Resolution (Å) (GS-FSC (0.143)						
masked (tight, corrected)	2.4	2.4	2.6	2.7	2.6	2.8
unmasked	3.1	3.1	3.5	3.9	3.6	4.3
EMDB ID	76336					
**Model refinement, composition, and validation** (AHA + E/E-tRNA) | PDB ID (12DM)						
**Model refinement**						
PDB ID	12DM					
Map-to-model cross correlation	0.82					
RMSD bond lengths (Å) [# > 4σ]	0.004 [0]					
RMSD bond angles (°) [# > 4σ]	0.600 [6]					
**Model composition**						
Non-hydrogen atoms	142,666					
Protein residues	6,562					
Nucleotide residues	4,266					
Ligand molecules	2 (AMP)					
**Model validation**						
MolProbity score	1.09					
Q-Score (mean)	0.63					
Atom inclusion	0.90					
Clash score	2.98					
C-beta outliers (%)	0.00					
Rotamer outliers (%)	0.04					
Ramachandran plot						
favored (%)	98.01					
allowed (%)	1.99					
outliers (%)	0.00					
Mean B-factor ($Å^2$)						
protein	33.12					
RNA	36.90					
ligands	32.29					

Appendix Table S2. Strains, plasmids and primers used in this work.

Strain	Genotype	Reference
***H. volcanii* DS2 (aBL3)**	-	Hartman *et al.*, 2010
***H. volcanii* H26 (aBL8)**	ΔpyrE2	Allers *et al.*, 2004
***H. volcanii* ΔAHA (aBL638)**	ΔpyrE2 ΔHVO_2384	This work
***H. volcanii* H26 pAL750 (aBL639)**	ΔpyrE2 pAL750	This work
***H. volcanii* ΔAHA pAL750 (aBL640)**	ΔpyrE2 ΔHVO_2384 pAL750	This work
***H. volcanii* ΔAHA pAL750-AHA (aBL641)**	ΔpyrE2 ΔHVO_2384 pAL750::HVO_2384	This work
***H. volcanii* H26-GFP (aJM191)**	ΔpyrE2 pyrE2::p27-msfGFP-mevR	This work

Plasmid	Genotype (description)	Reference
**pAL750 (eTR37)**	pAL750 (empty vector; Pxyl pro-moter)	(Rados et al., 2023)
**pAL750-AHA (eZC128)**	pAL750::HVO_2384 (AHA expres-sion under Pxyl)	This work
**pTSD1 (eTR68)**	pTSD1 (knock out generation in Hfx. volcanii)	(Rados et al, 2025)
**pTSD1-AHA (eDS1)**	pTSD1::HVO_2384u-HVO_2384d (ΔAHA generation)	This work
**pBlue-GFP (eJM53)**	pBlue-pyrE2::p27-msfGFP-mevR (msfGFP expression)	This work

Primers	Sequence (5'→3')	Use
**pTSD1_HVO_2384-up-fw**	CCGAAAAGTGCCACCTTCGTCGAAGACGACGTTC	eDS1 generation
**HVO_2384-up-rv**	CATCCTTACATACAACGCCG	eDS1 generation
**HVO_2384-up-HVO_2384-down-fw**	CGGCGTTGTATGTAAGGATGCAAGCTCAACGAGCTGTAAG	eDS1 generation
**pTSD1_HVO_2384-down-rv**	CGAGGGGTTTTATCCACGGTGGCGACGACTATCAC	eDS1 generation
**Pxyl_HVO_2384-fw**	GCTGGTAATGAGGATACTGCAATGGATATTGCTGATATCGCC	eZC128 generation
**Pxyl_HVO_2384-rv**	GGCCGCTCTAGAACTAGTTTACAGCTCGTTGAGCTTG	eZC128 generation
**pBlue-pyrE2-up-fw (oBL136)**	CCGAAAAGTGCCACCTCGGCGGTAGAAGTACG	eJM53 generation
**pyrE2-up-rv (oBL87)**	CTTGTTTCGAGAGGGTTCAG	eJM53 generation
**p27_pyrE2-up-fw (oJM212)**	CTGAACCCTCTCGAAACAAGACCCGCCGACTCGGCGT	eJM53 generation
**msfGFP_mevR-rv (oJM213)**	TCTTCCGCTTCCTCGTCATTTGTAAAGTTCATCCATTCCAT	eJM53 generation
**pyrE2-mevR-fw (oBL88)**	CTGAACCCTCTCGAAACAAGCGAGGAAGCGGAAGAG	eJM53 generation
**mevR_rv_pyrE2 (oJM160)**	AGTGAATTAGTCACAAGCGTGTACATGGGAGGGGATGG	eJM53 generation
**pyrE-down-fw (oHV42)**	TACACGCTTGTGACTAATTCACT	eJM53 generation
**pyrE2-down-rv (oBL137)**	GCTGGCCTTTTGCTCAGTGGCGGATTCGATGTAG	eJM53 generation
**Pscript-fw (oBL105)**	TGAGCAAAAGGCCAGC	eJM53 generation
**pTA131-Pamp-rv (oBL97)**	AGGTGGCACTTTTCGG	eJM53 generation

APPENDIX FIGURES

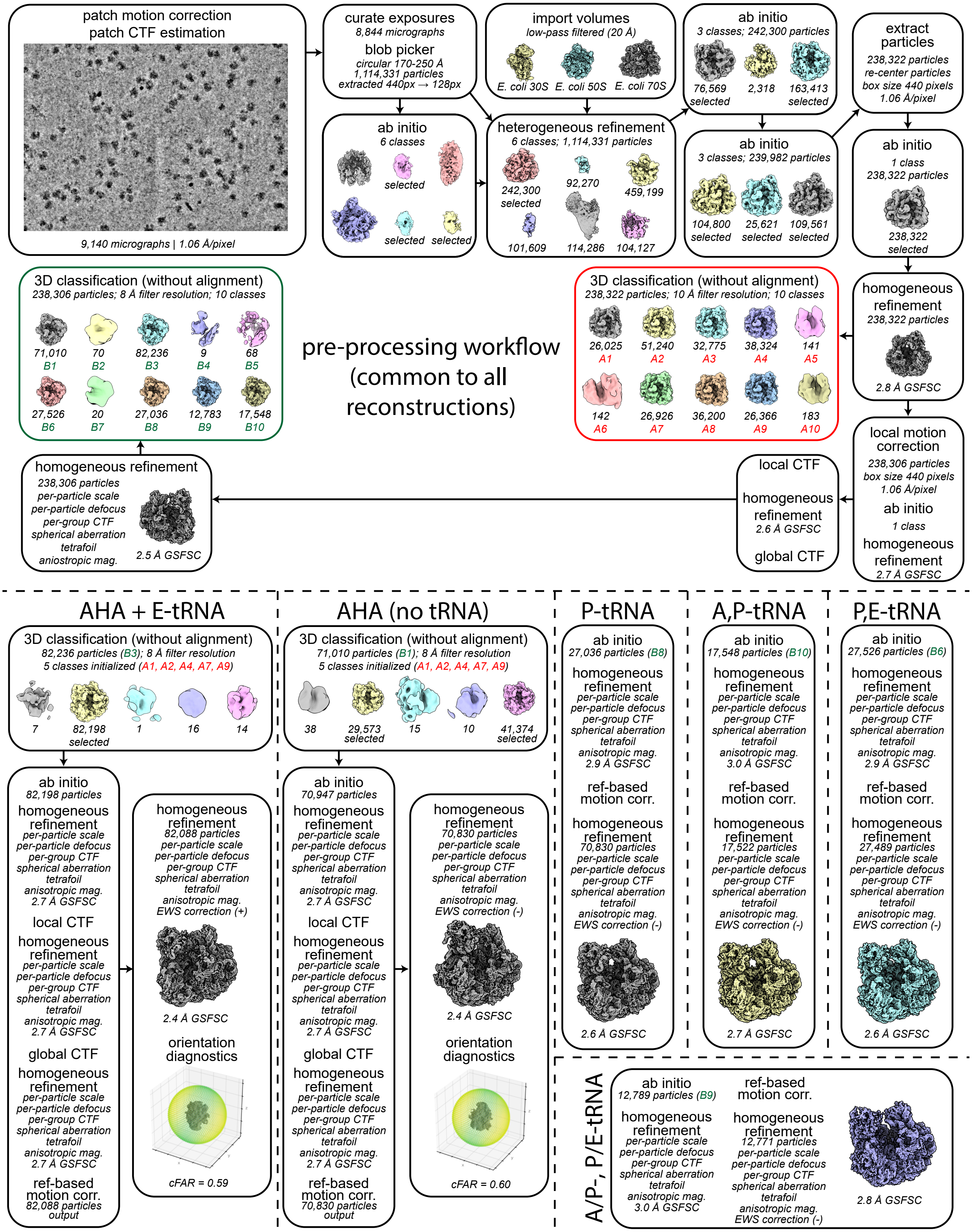

Appendix Figure S1. CryoEM image processing workflow. Schematic of the cryoSPARC-based (Punjani *et al*., 2017) cryoEM data processing pipeline used to obtain high-resolution consensus 70S ribosome reconstruction (top) and to classify relevant substates (bottom). Job names, particle counts, key metadata, and any non-default parameters are noted.

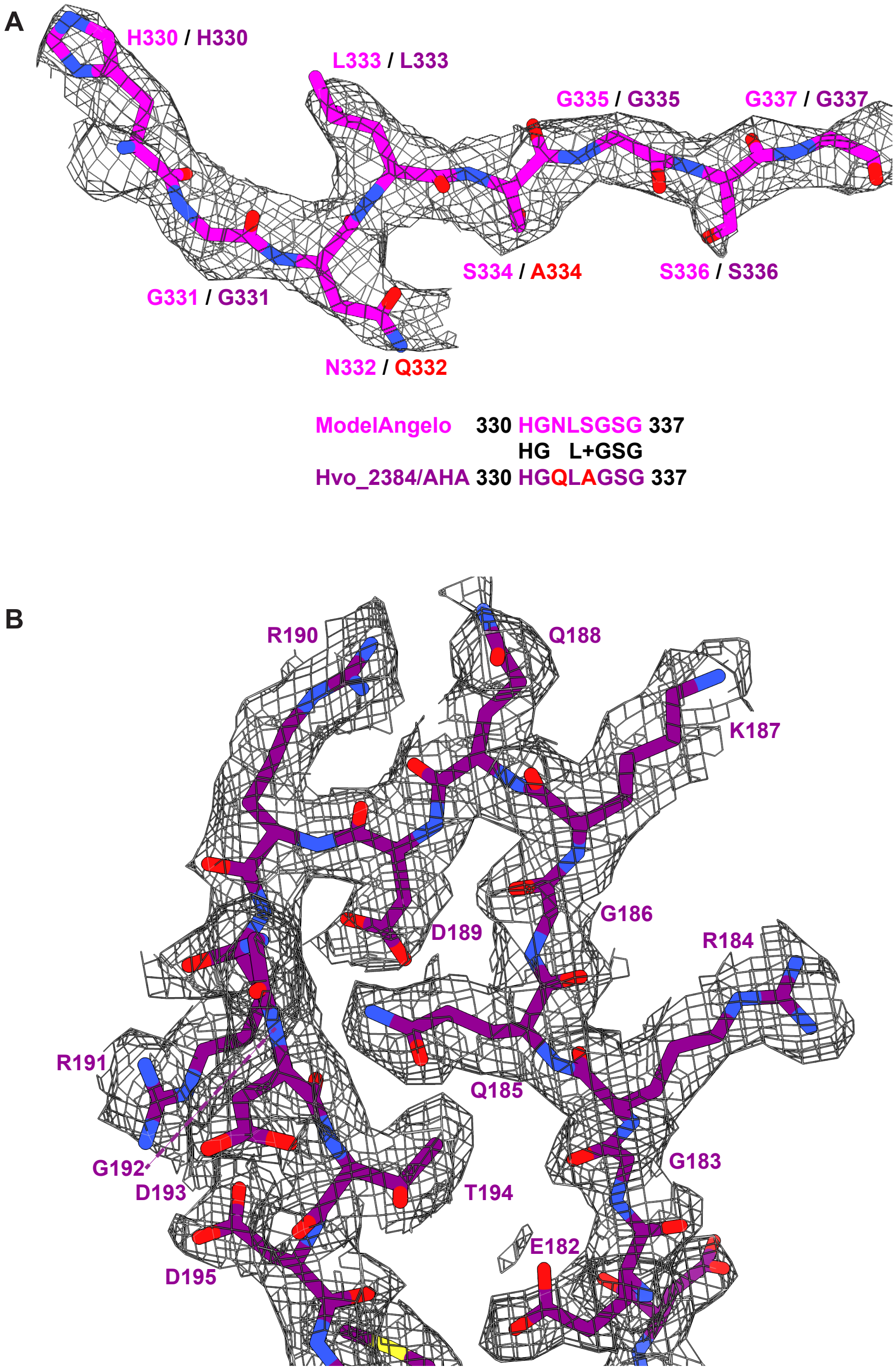

Appendix Figure S2. Automated atomic modeling-based identification of HVO_2384 (AHA) bound to a translationally inactive ribosome and final refined atomic model.

(A) CryoEM density (black mesh) from the AHA + E/E-site tRNA map (Figure 1F, Table S1) is shown with the unrefined, ModelAngelo-built atomic model docked into the map (top). Below, the amino acid sequence inferred by ModelAngelo (pink) is aligned with the annotated HVO_2384 sequence in this region (purple). Residue mismatches (highlighted in red) reflect positions where amino acids with chemically similar side-chains cannot be unambiguously distinguished at this resolution when the sequence is not provided to the algorithm.

(B) CryoEM density (black mesh) from the AHA + E/E-site tRNA map is presented with the refined atomic model of the AHA loop (residues 182-195) that inserts into the peptidyl transferase center. Oxygen, nitrogen, and sulfur atoms are displayed in red, blue, and yellow. Carbons are represented in pink for the unrefined ModelAngelo-built atomic model in (A) or purple for the refined model of AHA in (B).

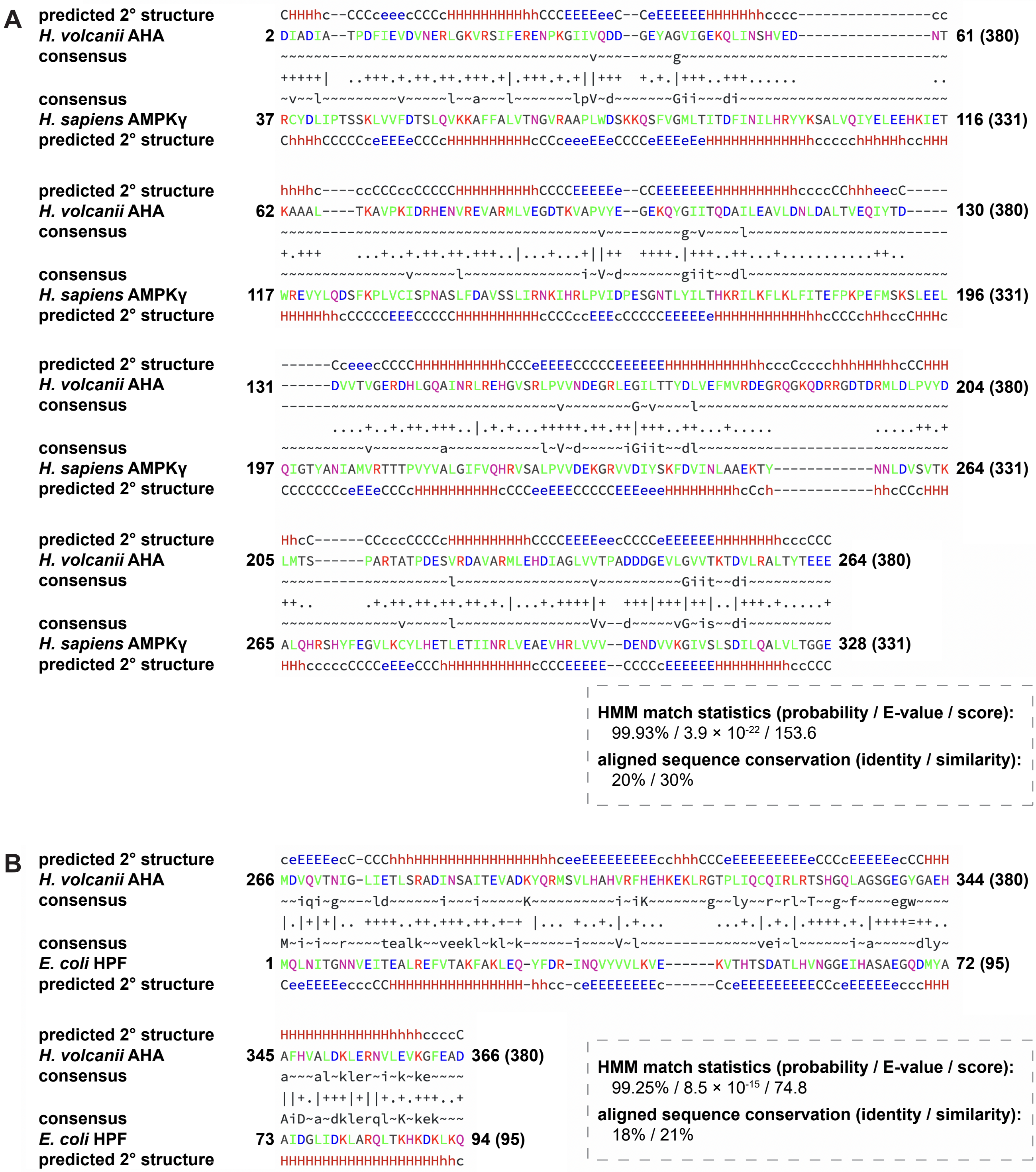

Appendix Figure S3. Archaeal AHA comprises an N-terminal CBS tetrad homologous to eukaryotic AMPKγ and a C-terminal HPF-like domain homologous to bacterial HPF. Homology between the N- and C-terminal domains of *Hfx. volcanii* AHA (HVO_2384) and **(A)** human AMPKγ (Uniprot: P54619) and **(B)** *E. coli* HPF (Uniprot: P0AFX0) was identified using HHpred (Zimmermann *et al.*, 2018), based on profile–profile comparison of their respective hidden Markov models (HMMs). Shown are the optimal HHpred alignments, with probabilities, E-values and scores indicated. Despite low pairwise sequence identity (20% for CBS-AMPKγ and 18% for HPF), the high HMM probabilities support deep evolutionary relationships. For the CBS comparison, the AHA N-terminal sequence was used to generate an HMM profile that was searched against a *Homo sapiens* profile database. For the HPF comparison, HMM profiles were constructed from provided multiple sequence alignments of archaeal AHA homologues (64 proteins) and bacterial HPF proteins (53 proteins). Residue numbering is indicated at the start and end of each sequence block. Consensus rows represent the most probable residue at each HMM position; uppercase and lowercase letters reflect the strength of conservation in the profile. Alignment symbols denote similarity inferred from the profile–profile comparison: | identical residues; + strong similarity; . weak similarity; blank, no detectable similarity. Amino acids are colored by physicochemical class (apolar, green; positively charged, red; negatively charged, blue; polar uncharged, magenta; small residues, black). Predicted secondary structure is shown above and below the sequences (helices, H/h, red; β-strands, E/e, blue; coils C/c, black; uppercase indicates higher confidence).

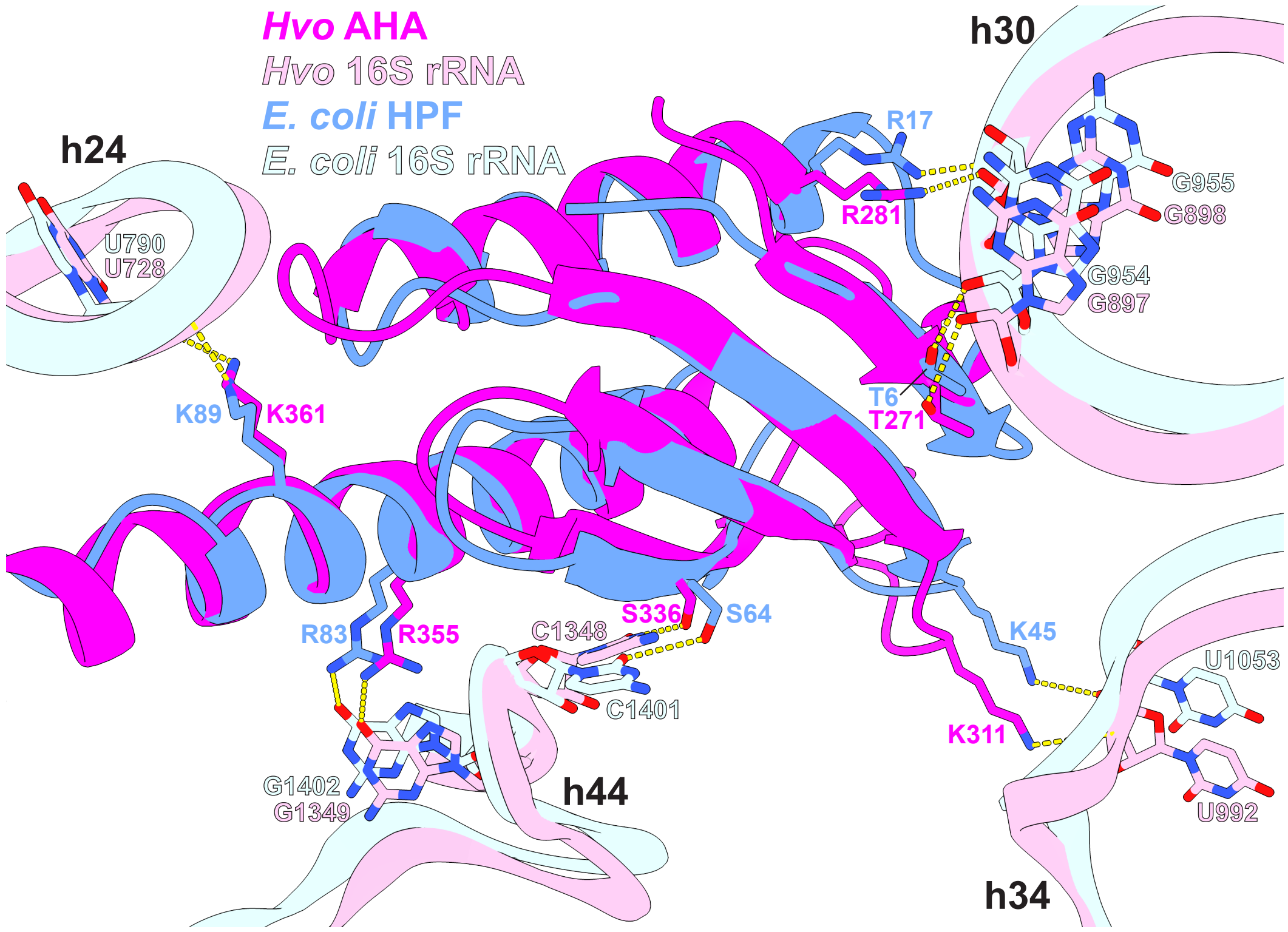

Appendix Figure S4. Conserved ribosomal recognition by archaeal and bacterial HPF domains. Structural comparison of archaeal (*Hfx. volcanii*, this work) and bacterial (*E. coli*, PDB 6Y69) HPF domains bound to the small ribosomal subunit highlighting a conserved set of interactions with 16S rRNA. Archaeal and bacterial HPFs and 16S rRNAs are colored following the legend. Equivalent residues that contact conserved 16S rRNA residues in each system are indicated, together with the corresponding 16S rRNA helix numbers.

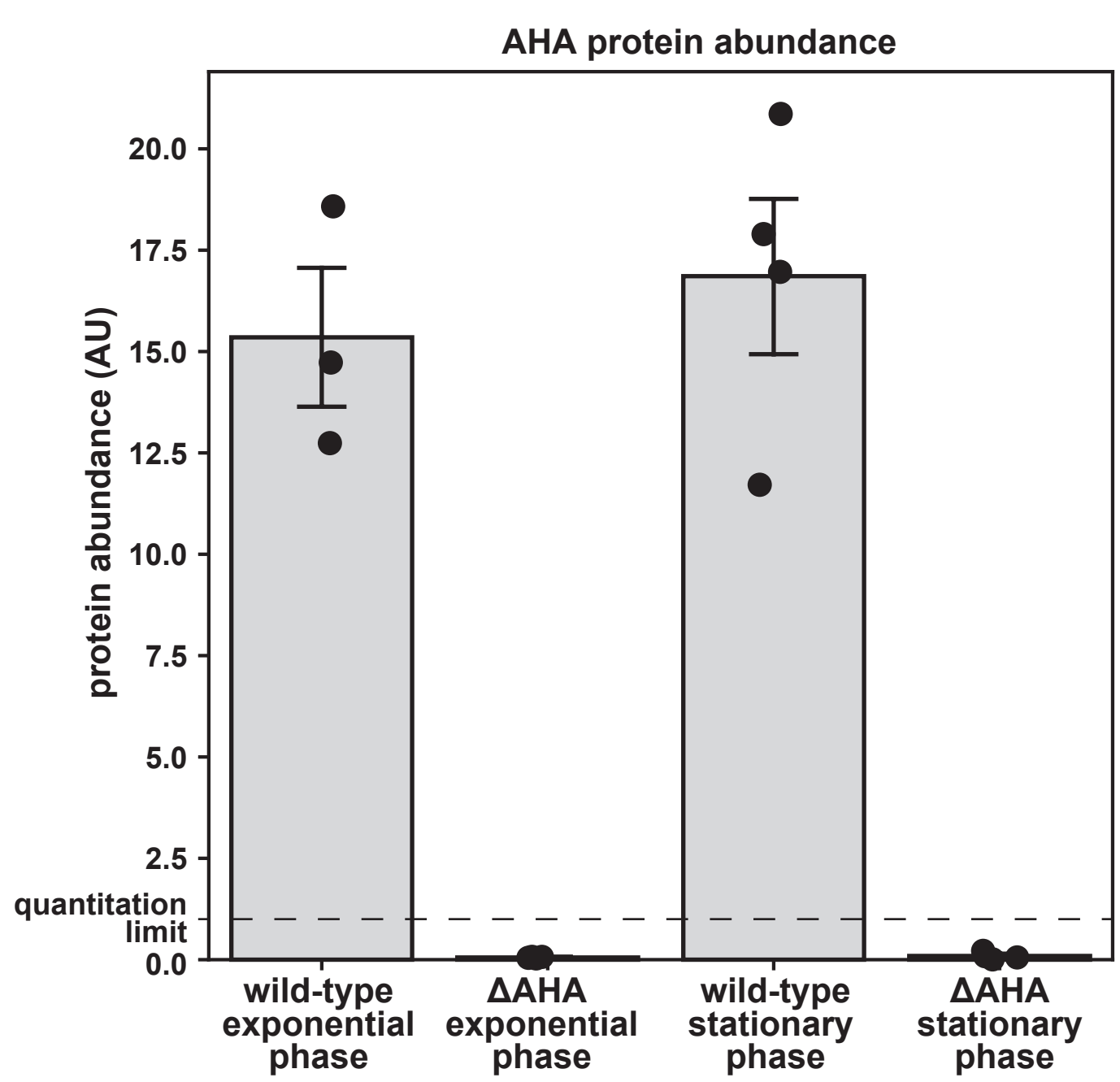

Appendix Figure S5. AHA expression is not regulated by growth phase. Quantitative mass spectrometry analysis of AHA protein abundance in wild-type and ΔAHA *Hfx. volcanii* cells during exponential and stationary phase growth. AHA peptides (n = 4) were robustly detected and quantified in wild-type cells at similar levels in both growth states, whereas AHA-derived peptides in the ΔAHA strain remained below the quantitation limit of the assay (dashed line). Data points represent individual biological replicates (n≥3); bars denote mean abundance ± standard error of the mean.

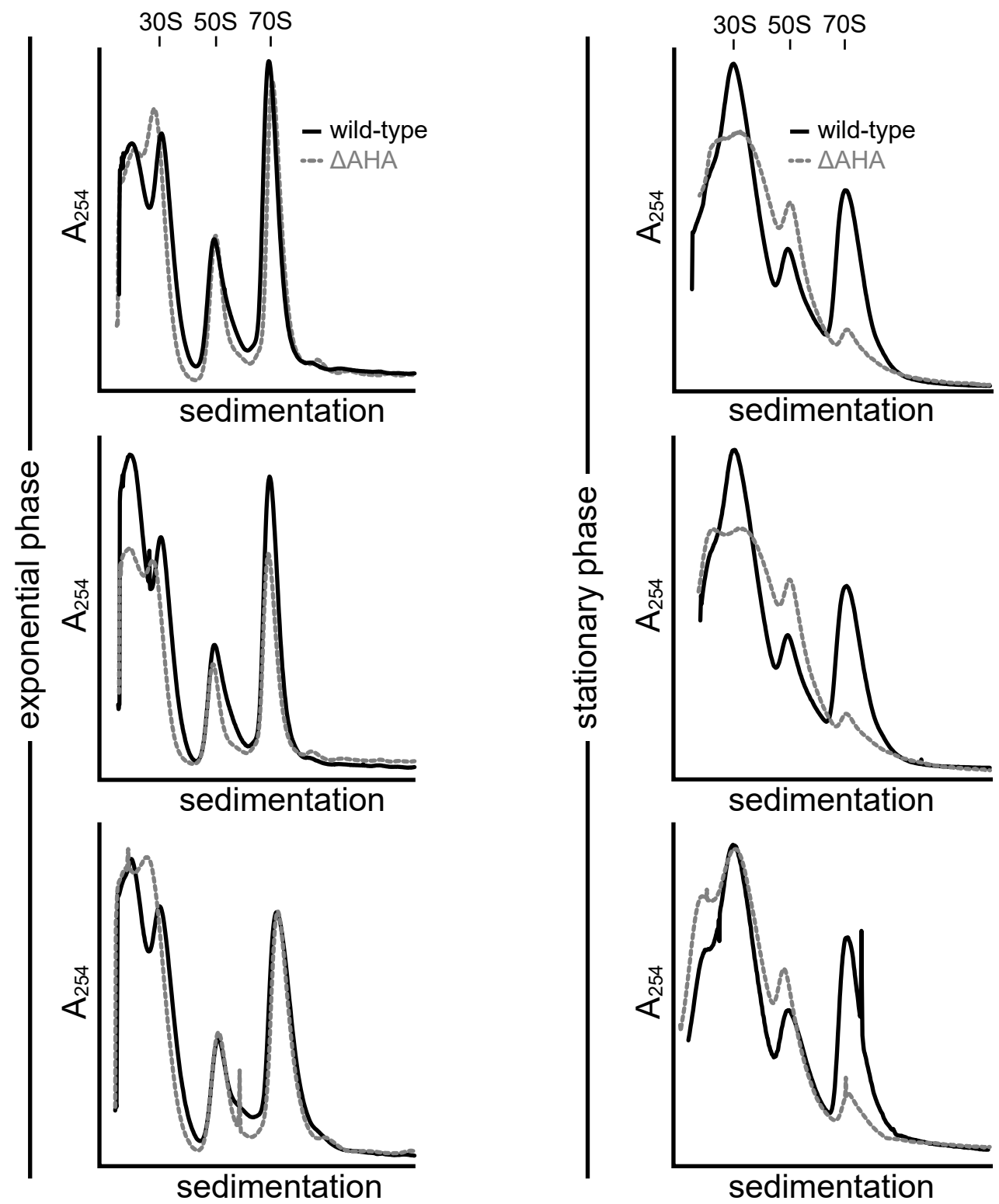

Appendix Figure S6. AHA preserves 70S ribosomes during stationary phase. Sucrose-gradient sedimentation profiles of wild-type (black) and ΔAHA (gray dotted) lysates prepared from exponential-phase (left) and stationary-phase (right) cultures. Rows show three technical replicates. Absorbance at 254 nm (A_{254}) is plotted as a function of sedimentation, with the migration positions of 30S, 50S, and 70S ribosomal species indicated.

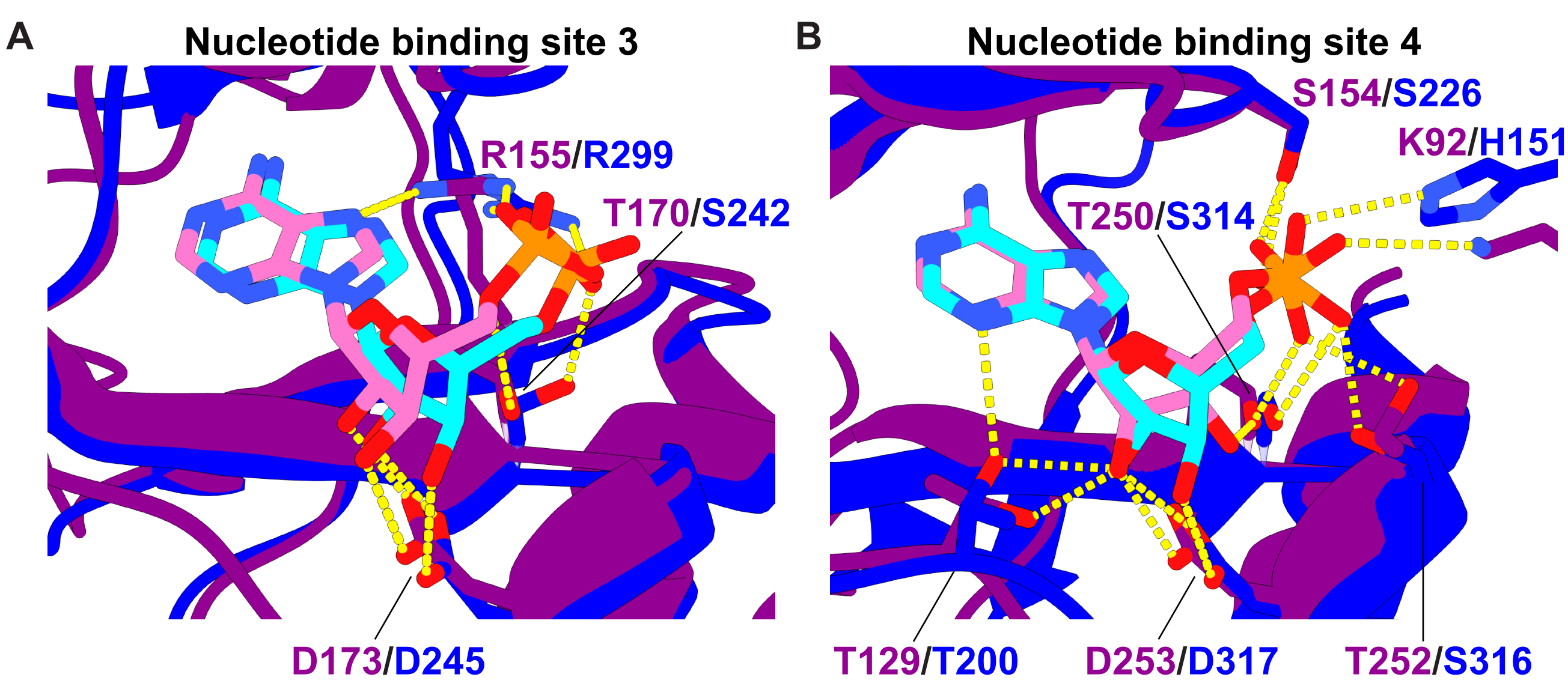

Appendix Figure S7. Comparison of the nucleotide binding sites in AHA and AMPKγ. Structural superposition of the AMP-binding pockets in AHA 4×CBS domain (protein chain and amino acid labels in purple; AMP carbon atoms in pink) and the eukaryotic AMPKγ subunit (PDB: 6B1U), with its protein and amino acid labels in dark blue, and AMP molecules in cyan. Equivalent or similar protein side chains forming hydrogen bonds or electrostatic interactions with the nucleotides are labeled, and interactions are indicated by yellow dashed lines. AHA's arginine (R) 155 and AMPKγ's R299 are not homologous residues as they are in different β-strands but similarly interact with the same AMP region. Oxygen, nitrogen, and phosphorus atoms are colored red, blue, and orange, respectively.

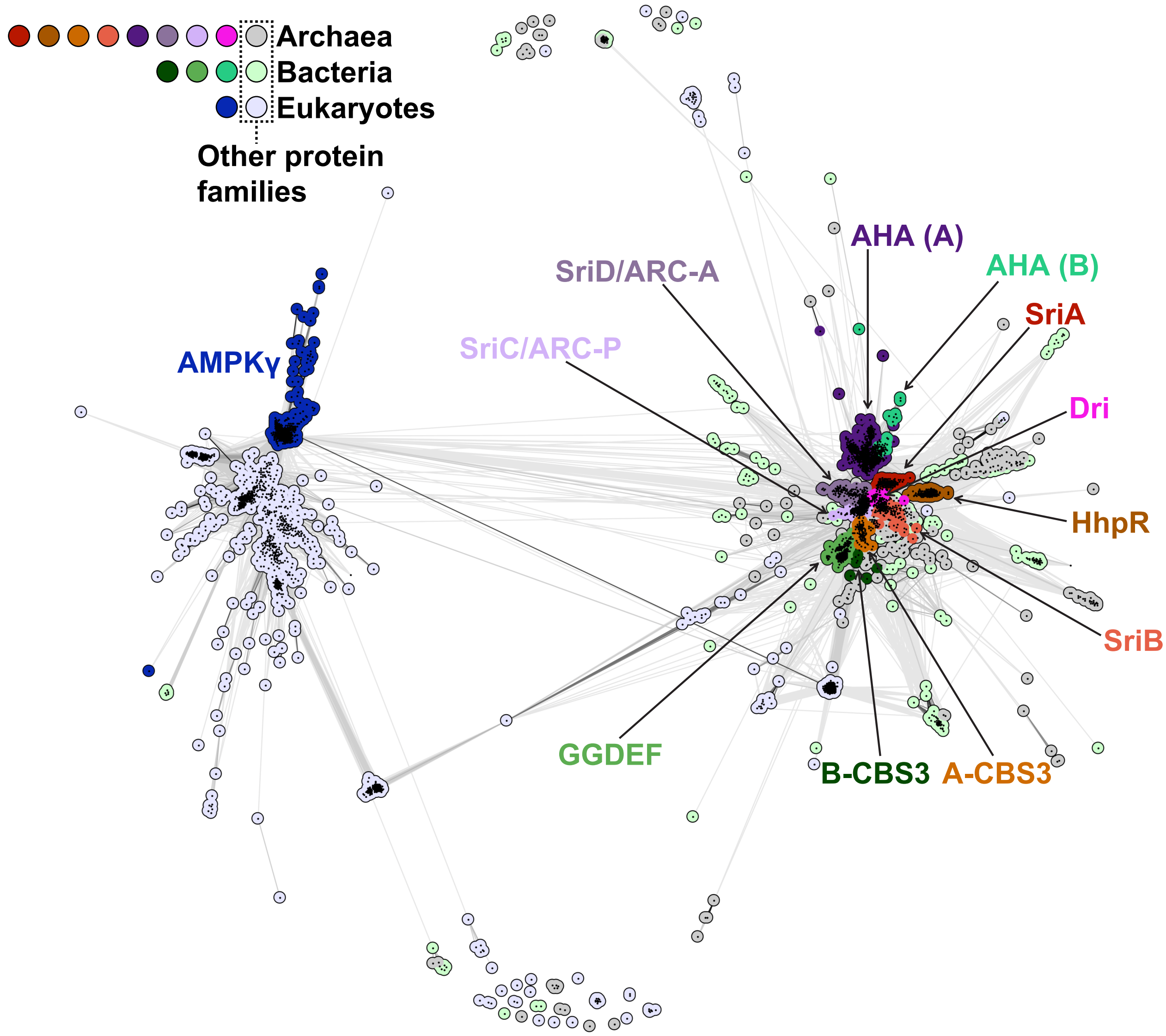

Appendix Figure S8. CLANS cluster-map analysis of the CBS-domain superfamily based on all-against-all pairwise sequence similarities. Each node represents an individual protein, and connecting lines reflect BLAST p-values, with darker lines indicating higher sequence similarity. Proteins are colored by taxonomic origin: bacteria (shades of green), eukaryotes (dark blue and lavender-blue), and archaea (all other colors). Protein families labeled: archaeal (A) AHA, Dri, SriA, SriB, SriC/ARC-P, SriD/ARC-A, HhpR and A-CBS3; bacterial (B) AHA homolog, GGDEF-containing 4×CBS fusion (GGDEF) and B-CBS3; eukaryotic AMPKγ.

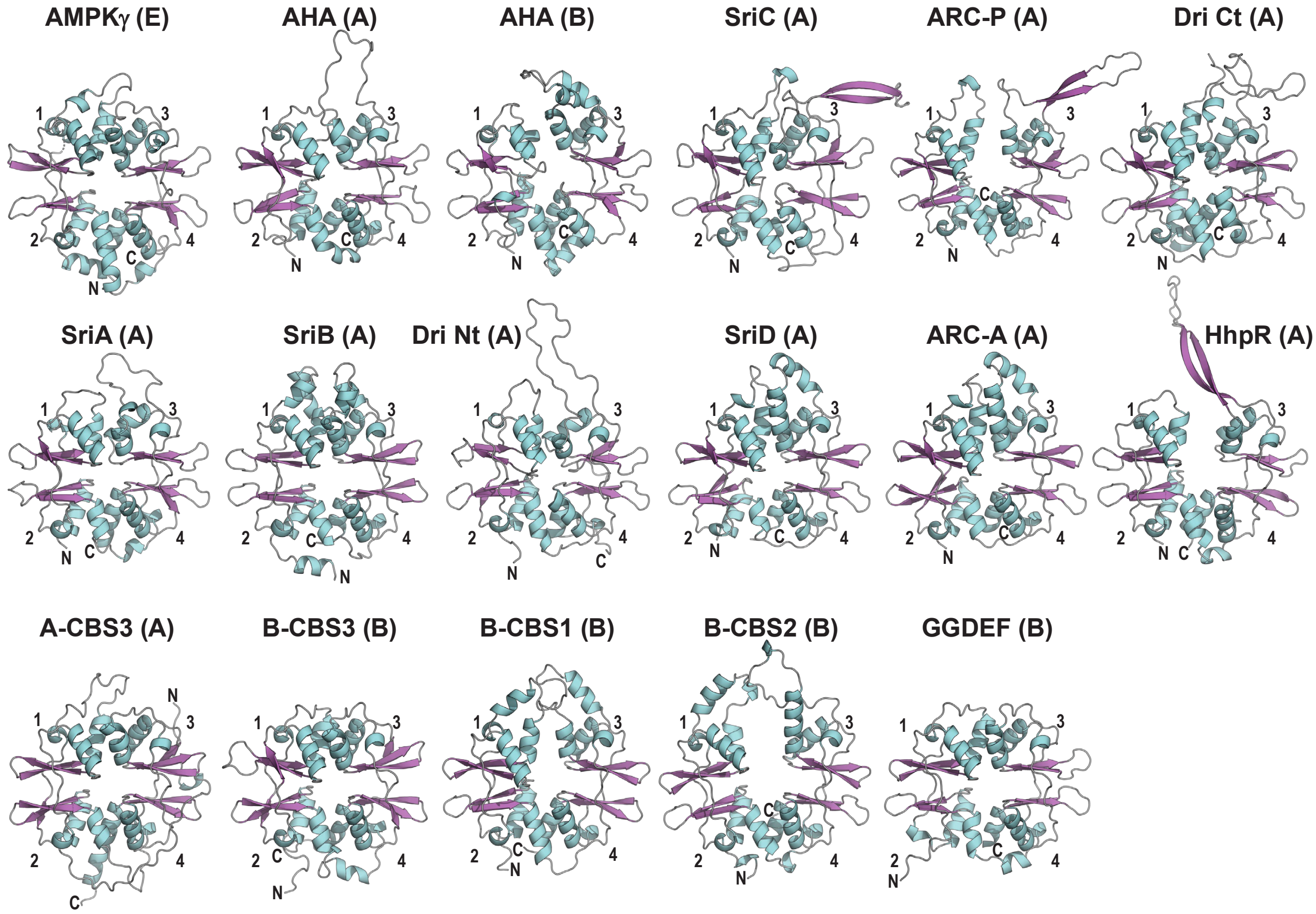

Appendix Figure S9. Structural comparison of members of the CBS-domain protein superfamily related to the N-terminal region of AHA. Representative CBS-containing proteins from eukaryotes (E), archaea (A), and bacteria (B) are shown [species; structural source (PDB or AlphaFold Database, AFDB) accession]: AMPKγ (Homo sapiens; PDB: 6B1U); AHA (*Hfx. volcanii*; cryoEM structure, this work); bacterial AHA homolog (*Candidatus Gottesmanbacteria bacterium*; AFDB: A0A1F5YVL3); SriA, SriC, SriD (*Methanosarcina acetivorans*; PDB: 9ZNF, 9ZNI, 9ZNI); SriB (*Methanosarcina acetivorans*; AFDB: Q8TH73); ARC-P and ARC-A (*Methanolobus sp. ZRKC1*; NCBI: WYM85633.1 and WYM85634.1; computed using AlphaFold3); Dri N-terminal (Nt) and C-terminal (Ct) domains, shown separately (*Pyrobaculum calidifontis*; PDB: 9E6Q and 9E7F); HhpR (*Pyrococcus yayanosii*; AFDB: F8AFU3); archaeal CBS3 (A-CBS3; miscellaneous Crenarchaeota group-1 archaeon SG8-32-3; AFDB: A0A0M0BVG1); bacterial CBS1 (B-CBS1; *Anaerolinea thermophila*; AFDB: E8N4C0); bacterial CBS2 (B-CBS2; *Deinococcus reticulitermitis*; AFDB: A0A1H6XD96); bacterial CBS3 (B-CBS3; *Candidatus Nitrospira nitrificans*; AFDB: A0A0S4LEU7); and GGDEF-containing 4×CBS fusion proteins (*Romeriopsis navalis*; AFDB: A0A928VUH7). The four CBS motifs within each protein (CBS1–CBS4) are labeled numerically. N- and C-termini are indicated for reference. α-helices are shown in teal and β-strands in pink. The long, extended disordered N-terminal region of SriB is omitted for clarity, whereas the N-terminal extension in A-CBS3 is shown.
