# Supplementary material for: A conserved archaeal protein with evolutionary links to bacterial ribosome hibernation and eukaryotic energy sensing
Source: EMBO J. 2026 Oct 2;45(21):7564–81. doi: 10.1038/s44318-026-00918-6 (PMC13728122; doi:10.1038/s44318-026-00918-6)
Supplement: Supplementary file 2 [file 44318_2026_918_MOESM2_ESM.pdf]

THE EMBO JOURNAL

A conserved archaeal protein with evolutionary links to bacterial ribosome hibernation and eukaryotic energy sensing

Diorge Souza, Mira May, Jackson Carrion, Vikram Alva, Alex Bisson, and Joseph Davis

Corresponding author(s): Joseph Davis (jhdavis@mit.edu) , Diorge Souza (diorge@mit.edu), Alex Bisson (bisson@iu.edu)

Review Timeline:

Submission Date:	30th Mar 26
Editorial Decision:	5th May 26
Revision Received:	4th Aug 26
Editorial Decision:	31st Aug 26
Revision Received:	9th Sep 26
Accepted:	14th Sep 26

Editor: Hartmut Vodermaier

Transaction Report:

(Note: With the exception of the correction of typographical or spelling errors that could be a source of ambiguity, letters and reports are not edited. Depending on transfer agreements, referee reports obtained elsewhere may or may not be included in this compilation. Referee reports are anonymous unless the Referee chooses to sign their reports.)

Prof. Joseph H Davis
Massachusetts Institute of Technology
Biology
77 Massachusetts Ave.
Cambridge, MA 02139

5th May 2026

Re: EMBOJ-2026-124255
A conserved archaeal factor linking bacterial ribosome hibernation and eukaryotic energy sensing

Dear Joey,

Thank you for submitting your manuscript on a new archaeal ribosome hibernation factor, AHA, to The EMBO Journal. I sent it to three expert referees, who have now provided the reports copied below. Since all of them consider the work highly interesting, we would be happy to pursue it further for publication, pending adequate revisions in response to the referees' comments.

Given that the majority of points raised are linked to analyses and presentation of the data, I hope you will be able to resubmit a revised manuscript within roughly one month. Detailed information on preparing, formatting and uploading a revised manuscript (incl. separate text and figure files, source data, various checklists) can be found below and in our Guide to Authors, and adhering to them as closely as possible shall greatly facilitate editorial processing upon resubmission. As always, our scooping protection policy means that competing work appearing elsewhere during that time will not affect our final decision on your study, but should any of the various cited preprints become formally published in the meantime, please make sure to cover/contrast/compare them adequately in the discussion section.

Please do not hesitate to contact me should you have any other questions regarding the reports or this decision. Thank you again for the opportunity to consider this work for The EMBO Journal, and I look forward to your revision.

With kind regards,

Hartmut

Hartmut Vodermaier, PhD
Senior Editor, The EMBO Journal
h.vodermaier@embojournal.org

*** PLEASE NOTE: All revised manuscript are subject to initial checks for completeness and adherence to our formatting guidelines. Revisions may be returned to the authors and delayed in their editorial re-evaluation if they fail to comply to the following requirements. As a first step please read our guidelines for revised submissions:
https://link.springer.com/journal/44318/submission-guidelines#cms-Revised-submissions

1) Every manuscript requires a Data Availability section (even if only stating that no deposited datasets are included). Primary datasets or computer code produced in the current study have to be deposited in appropriate public repositories prior to resubmission, and reviewer access details provided in case that public access is not yet allowed.

2) Each figure legend must specify
- size of the scale bars that are mandatory for all micrograph panels
- the statistical test used to generate error bars and P-values
- the type error bars (e.g., S.E.M., S.D.)
- the number (n) and nature (biological or technical replicate) of independent experiments underlying each data point
- Figures may not include error bars for experiments with n<3; scatter plots showing individual data points should be used instead.

3) Revised manuscript text (including main tables, and figure legends for main and EV figures) has to be submitted as editable text file (e.g., .docx format). We encourage highlighting of changes (e.g., via text color) for the referees' reference.

4) Each main and each Expanded View (EV) figure should be uploaded as individual production-quality files (preferably in .eps, .tif, .jpg formats). For suggestions on figure preparation/layout, please refer to our Figure Preparation Guidelines:
https://media.springernature.com/original/springer-cms/rest/v1/content/27825798/data/v1

5) Point-by-point response letters should include the original referee comments in full together with your detailed responses to them (and to specific editor requests if applicable), and also be uploaded as editable (e.g., .docx) text files.

6) Please complete our Author Checklist, and make sure that information entered into the checklist is also reflected in the manuscript; the checklist will be available to readers as part of the Review Process File.

7) All authors listed as (co-)corresponding need to deposit, in their respective author profiles in our submission system, a unique ORCiD identifier linked to their name. Please see our Guide to Authors for detailed instructions.

8) Please note that supplementary information at EMBO Press has been superseded by the 'Expanded View' for inclusion of additional figures, tables, movies or datasets; with up to five EV Figures being typeset and directly accessible in the HTML version of the article.

9) To facilitate reproducibilty and cross-laboratory adoption of methodologies, please structure the Materials & Methods section as outlined in our guide to authors, including a completed Reagents and Tools Table.

10) Digital image enhancement is acceptable practice, as long as it accurately represents the original data and conforms to community standards. If a figure has been subjected to significant electronic manipulation, this must be clearly noted in the figure legend and/or the 'Materials and Methods' section. The editors reserve the right to request original versions of figures and the original images that were used to assemble the figure. Finally, we generally encourage uploading of numerical as well as gel/blot image source data.

At EMBO Press we ask authors to provide source data for the main manuscript figures. You will receive a separate email with instructions for providing source data with your revised manuscript, including how to upload and organize the files.

In the interest of ensuring the conceptual advance provided by the work, we recommend submitting a revision within 3 months (3rd Aug 2026). Please discuss the revision progress ahead of this time with the editor if you require more time to complete the revisions. Use the link below to submit your revision:

Link Not Available

Referee #1:

Living cells, including archaea, possess a remarkable superpower - the ability to enter states of suspended animation in which they show no overt signs of growth yet retain full viability. This allows them to survive for exceedingly long periods ranging from days to millions of years in the complete absence of nutrients, at temperatures incompatible with growth, or under other extreme environmental conditions. We humans lack these abilities, and we stand to learn a great deal from microorganisms about how to preserve living cells in hostile environments - knowledge with far-reaching applications in medicine, agriculture, space travel, and beyond.

Previous studies in bacteria and some eukaryotes have shown that this remarkable resilience of living cells in the face of environmental stress is driven to a large extent by a small group of proteins known as hibernation factors. It has been demonstrated that these proteins bind to ribosomes and protect them from degradation. This allows cells to preserve their most metabolically expensive molecules required for growth in intact form, and to rapidly resume growth once conditions improve or cells adapt to new environments.

In this outstanding study by the Joseph Davis lab, Souza et al. report the identification and characterisation of one of these "hibernation superpower" proteins - protein AHA (AMPKγ-HPF from Archaea), a broadly conserved archaeal ribosome hibernation factor discovered by applying cryoEM directly to crude lysates of Haloferax volcanii cells entering stationary phase. Their structural analysis of this factor bound to the ribosome reveals that AHA is a two-domain protein: its C-terminal domain is homologous to bacterial HPF and occupies the same site on the small ribosomal subunit, occluding the A- and P-tRNA binding sites; its N-terminal domain comprises four CBS motifs that bind the large subunit and insert a loop into the peptidyl transferase centre.
Furthermore, the authors observe AMP molecule bound to the CBS domain at positions equivalent to the canonical nucleotide-sensing sites of eukaryotic energy-sensing protein AMPKγ, suggesting that-similarly to AMPKγ, AHA activity may be regulated by cellular energy status. Consistent with this idea, the authors show that deletion of AHA in H. volcanii results in reduced viability of this archaeal organism in stationary phase, impaired regrowth, decreased competitive fitness, and coordinated depletion of ribosomal proteins, consistent with a role of AHA in ribosome protection during hibernation.

Their robust phylogenetic analyses across ~7,000 archaeal genomes demonstrate that HPF-containing proteins are broadly distributed across archaeal phyla, and maximum-likelihood trees recover bacterial and archaeal HPF sequences as two deeply divergent clades with maximal statistical support, consistent with an origin in the last universal common ancestor. CBS-domain phylogenetics further supports the shared evolutionary origin of the hibernation factor AHA and the eukaryotic energy sensing protein AMPKγ.

As an expert in ribosome hibernation, I find this discovery profoundly impactful as it helps explain how archaeal organisms survive prolonged periods in nutrient-deprived environments. I cannot support its publication more strongly.

Major concerns:
I have no major concerns.

Minor concerns:
While the culture of peer review tends toward requesting ever more experiments and analyses - rarely improving manuscripts while delaying important science - I would instead recommend only that the manuscript be streamlined by removing a few unsupported speculative statements.

Specifically, I suggest them to remove these words "thereby silencing translation" from their abstract and corresponding parts of their manuscript because the characterization of this protein as a translation inhibitor is not supported by the data. Of course, it is self-evident that ribosomes bound to a hibernation factor cannot support active translation, but this does not necessarily mean that the factor itself acts as an inhibitor. For instance, many translation initiation factors occlude the binding sites for mRNA and tRNA, yet no one would call them inhibitors of protein synthesis solely based on this structural overlap. Furthermore, it seems highly likely to me that in the absence of this factor, cells can downregulate protein synthesis equally efficiently in response to stationary phase, indicating that other mechanisms are responsible for this inhibition.

Secondly, I suggest rephrasing the claim "thus linking archaeal and eukaryotic energy sensing" because it is potentially misleading, as it implies that the newly discovered hibernation factor is functionally involved in energy sensing, for which no supporting data are provided. I would suggest revising this statement (as well as the associated statements in the text including the title) in order to clarify that the intended meaning is evolutionary linking rather functional or structural linking- for example, "revealing the shared evolutionary origin of this hibernation factor and the eukaryotic nutrient sensing protein AMPKγ" - which is both factually accurate and equally compelling.

Having said that, I find this work outstanding and complete, and cannot support its publication more strongly - it is not every day that one discovers a key ribosomal partner that helps cells survive prolonged periods of metabolic inactivity. I foresee that this work will be one of the cornerstones of our understanding of how archaeal cells preserve their most metabolically expensive macromolecular assemblies during periods of persistent stress.

Referee #2:

Souza et al. use cryo-EM analysis of archaeal cell lysates grown to stationary phase to examine ribosomes. They identify AHA, a ribosome-associated factor that binds across the ribosomal subunits, occludes the mRNA channel and tRNA-binding sites, and functions as a ribosome hibernation factor. AHA is composed of two modules: a C-terminal domain homologous to the bacterial hibernation-promoting factor (HPF), and an N-terminal CBS tetrad domain related to the eukaryotic energy sensor AMPKγ, which is bound to two AMP molecules in the cryo-EM maps. Phylogenetic analysis shows broad conservation of AHA-related proteins across archaea and suggests evolutionary links between the C-terminal HPF-like domain and bacterial hibernation factors, as well as between the CBS domain and eukaryotic energy-sensing proteins. The authors also show that AHA is required for cell survival during stationary phase and for efficient recovery upon nutrient restoration, and that loss of AHA alters the stationary-phase proteome. The observation that AMP binds within conserved pockets of the AHA CBS domain further suggests a mechanism by which ribosome hibernation may be coupled to cellular energy status.

This is an interesting study that provides important structural and functional insight into archaeal ribosome regulation. It also proposes a new connection between translational control and energy sensing across domains of life. There are several points however that the authors should address prior to publication.

Specific comments:

1. Did the authors attempt polysome gradient profiling of wild-type and ΔAHA cells across different growth stages, such as exponential phase, transition into stationary phase, stationary phase, and recovery? This experiment would help establish whether loss of AHA alters ribosome populations or translational states.

2. The MS data show depletion of more than 30 ribosomal proteins, predominantly from the large ribosomal subunit rather than

the small subunit. This is interesting, especially given that some studies in eukaryotic systems have reported preferential degradation of the small subunit under starvation conditions. One possibility is that loss of AHA causes or exacerbates a ribosome assembly defect. Polysome profiling would be useful here as well, as it could reveal changes in 50S/30S/70S populations in ΔAHA cells compared with wild type.

3. Related to the MS analysis, it would be informative to test whether ribosomal RNA levels on agarose gels for example, particularly the large-subunit rRNA, are similarly affected in ΔAHA cells. This could help assess if AHA protect the ribosomal RNA from degradation under stress conditions.

4. The cryo-EM analysis was performed using lysates from cells cultured in semi-defined media until stationary phase, and the data suggest that AHA responds to low-energy states. However, because translation is directly dependent on GTP hydrolysis, it would be useful for the authors to discuss why archaea may use an ATP/AMP-sensing factor to regulate ribosome hibernation. For comparison, bacterial translational shutdown is often linked to alarmone-based stress signaling. This point could be discussed more explicitly.

5. Because multiple nutrients may become limiting during stationary phase, did the authors examine defined nutrient-depletion conditions to determine which specific limitations affect AHA levels or ribosome association? This would help clarify whether AHA responds broadly to stationary phase or to particular metabolic or nutrient cues.

6. None of the figures show clear fits of the AHA atomic model into the corresponding cryo-EM density in either the main or supplementary figures. The exception is Figure S2, which shows density for only approximately seven residues. The authors should consider including additional model-to-density views to better support assignment and modeling of this factor. The structural close-ups as well are somewhat busy and could benefit from simplification.

Referee #3:

This paper reports the discovery of a Ribosome hibernation factor in achaea that represents an evolutionary link between engery sensing in eukaryotes and ribosme hibernation in prokaryotes. I find this paper very interesting, technically well executed and suitable for the general audience of EMBO.

The authors discover this new protein using a particularly interesting strategy of using lysates of stationary phase cells. I found this quite exciting. I also find the evolutionary implications very interesting and impactful. Perhaps the only deficiency of this paper is a lack of in vitro characterization, but I think that's quite clearly for future work and not needed for this study.

I am no expert no the cryo-electron microscopy and cannot comment on the rigor of these analyses. My expertise lies in phylogenetics and I have a few comments about the phylogenies in this paper. These are in general inferred carefully, but some of the interpretation needs clarification:

The first problem is that the authors infer a phylogeny of the HPF domain, in which they see a clear split between archaea and bacteria. They take this to indicate that this family originated in LUCA. While this pattern is suggestive, the authors have not ruled out a single, cross-domain horizontal transfer. To do this, they would need to show that the branching order within each domain is approximately what we expect from our understanding of the tree of life. I could not verify this from figure 2H.

In Figure 5 the authors then infer an unrooted tree of the CBS domain module of this new protein. Was puzzled by some of the descriptions in the text that went along with this phylogeny. For example, the authors state that AHA likely evolved from SriA. I don't understand this inference. No possible root of this tree would clearly place SriA inside AHA. Most roots would simply make them sister groups, with AHA closer to their shared node in terms of branch lnegths than SriA. Could the authors clarify their text? To be clear, this has no bearing on the impact of their paper.

Similarly, the authors also state that AMPK gamma evolved with the archaeal CBS3 family. The situation is exactly as for the two proteins mentioned above: Unless the root of the entire tree is within CBS3, Ampk gamma is a sister group to CBS3 and doesn't emerge from within it. Again, the authors need to clarify these sentences.

Response to the Editor and Reviewers

Manuscript: EMBOJ-2026-124255

Original title: *A conserved archaeal factor linking bacterial ribosome hibernation and eukaryotic energy sensing*

Revised title: *A conserved archaeal protein with evolutionary links to bacterial ribosome hibernation and eukaryotic energy sensing*

We sincerely thank the Editor, Dr. Hartmut Vodermaier, and the three reviewers for their careful evaluation of our manuscript and for their highly positive and constructive comments.

In the revised manuscript, we have carefully addressed each comment raised by the reviewers by including additional experiments, new supplementary figures, and by clarifying several evolutionary interpretations. We have also amended the text, as directed. We believe these changes have substantially strengthened the manuscript.

Below we provide a detailed point-by-point response. Reviewer comments are reproduced, followed by our responses. All modifications have been incorporated into the revised manuscript and highlighted in the tracked-changes version.

Referee #1

Living cells, including archaea, possess a remarkable superpower - the ability to enter states of suspended animation in which they show no overt signs of growth yet retain full viability. This allows them to survive for exceedingly long periods ranging from days to millions of years in the complete absence of nutrients, at temperatures incompatible with growth, or under other extreme environmental conditions. We humans lack these abilities, and we stand to learn a great deal from microorganisms about how to preserve living cells in hostile environments - knowledge with far-reaching applications in medicine, agriculture, space travel, and beyond.

Previous studies in bacteria and some eukaryotes have shown that this remarkable resilience of living cells in the face of environmental stress is driven to a large extent by a small group of proteins known as hibernation factors. It has been demonstrated that these proteins bind to ribosomes and protect them from degradation. This allows cells to preserve their most metabolically expensive molecules required for growth in intact form, and to rapidly resume growth once conditions improve or cells adapt to new environments.

In this outstanding study by the Joseph Davis lab, Souza et al. report the identification and characterisation of one of these "hibernation superpower" proteins - protein AHA (AMPKγ-HPF from Archaea), a broadly conserved archaeal ribosome hibernation factor discovered by applying cryoEM directly to crude lysates of Haloferax volcanii cells entering stationary phase. Their structural analysis of this factor bound to the ribosome reveals that AHA is a two-domain protein:

its C-terminal domain is homologous to bacterial HPF and occupies the same site on the small ribosomal subunit, occluding the A- and P-tRNA binding sites; its N-terminal domain comprises four CBS motifs that bind the large subunit and insert a loop into the peptidyl transferase centre.

Furthermore, the authors observe AMP molecule bound to the CBS domain at positions equivalent to the canonical nucleotide-sensing sites of eukaryotic energy-sensing protein AMPKγ, suggesting that-similarly to AMPKγ, AHA activity may be regulated by cellular energy status. Consistent with this idea, the authors show that deletion of AHA in H. volcanii results in reduced viability of this archaeal organism in stationary phase, impaired regrowth, decreased competitive fitness, and coordinated depletion of ribosomal proteins, consistent with a role of AHA in ribosome protection during hibernation.

Their robust phylogenetic analyses across ~7,000 archaeal genomes demonstrate that HPF-containing proteins are broadly distributed across archaeal phyla, and maximum-likelihood trees recover bacterial and archaeal HPF sequences as two deeply divergent clades with maximal statistical support, consistent with an origin in the last universal common ancestor. CBS-domain phylogenetics further supports the shared evolutionary origin of the hibernation factor AHA and the eukaryotic energy sensing protein AMPKγ.

As an expert in ribosome hibernation, I find this discovery profoundly impactful as it helps explain how archaeal organisms survive prolonged periods in nutrient-deprived environments. I cannot support its publication more strongly.

Major concerns: I have no major concerns.

We thank Referee #1 for the positive assessment of our work. We greatly appreciate the thoughtful suggestions, which primarily concern improving the precision of our conclusions and clarifying several interpretations. We have addressed each point below.

Minor concerns: While the culture of peer review tends toward requesting ever more experiments and analyses - rarely improving manuscripts while delaying important science - I would instead recommend only that the manuscript be streamlined by removing a few unsupported speculative statements.

Specifically, *I suggest them to remove these words "thereby silencing translation" from their abstract* and corresponding parts of their manuscript because the characterization of this protein as a translation inhibitor is not supported by the data. Of course, it is self-evident that ribosomes bound to a hibernation factor cannot support active translation, but this does not necessarily mean that the factor itself acts as an inhibitor. For instance, many translation initiation factors occlude the binding sites for mRNA and tRNA, yet no one would call them inhibitors of protein synthesis solely based on this structural overlap. Furthermore, it seems highly likely to me that in the absence of this factor, cells can downregulate protein synthesis equally efficiently in response to stationary phase, indicating that other mechanisms are responsible for this inhibition.

We agree with the reviewer that our structural and genetic data do not directly establish that AHA actively inhibits translation. Throughout the manuscript, we now describe AHA as a ribosome

hibernation factor whose binding is structurally incompatible with active translation, rather than as a demonstrated translation inhibitor. Specifically, we have revised the Abstract (page 2: "AHA bound across ribosomal subunits, occluding the mRNA channel and tRNA binding sites, supporting its role in ribosome hibernation"), Introduction (page 4: "consistent with a role in translational hibernation"), and Discussion (page 16: "These interactions are fundamentally incompatible with translation, indicating that AHA, like bacterial HPF, functions as a ribosome hibernation factor that stabilizes inactive ribosomes during energy limitation") to avoid wording that could be interpreted as claiming direct biochemical inhibition of translation.

Secondly, I suggest rephrasing the claim *"thus linking archaeal and eukaryotic energy sensing" because it is potentially misleading, as it implies that the newly discovered hibernation factor is functionally involved in energy sensing, for which no supporting data are provided.* I would suggest revising this statement (as well as the associated statements in the text including the title) in order to clarify that the intended meaning is evolutionary linking rather functional or structural linking- for example, "revealing the shared evolutionary origin of this hibernation factor and the eukaryotic nutrient sensing protein AMPKγ" - which is both factually accurate and equally compelling.

We appreciate the reviewer's thoughtful suggestion and agree that the data presented support an evolutionary connection between AHA and the eukaryotic AMPK pathway, and a functional implication in energy sensing, without direct demonstration. We have therefore revised the Title, Abstract (page 2: "supporting a shared evolutionary origin of the archaeal CBS-tetrad and the AMPKγ family. Together, these findings uncover a widespread archaeal ribosome hibernation factor and reveal an evolutionary connection between prokaryotic ribosomal hibernation and eukaryotic energy sensing"), and Discussion (page 17: "AHA provides direct structural and phylogenetic evidence supporting an evolutionary relationship between prokaryotic ribosome hibernation and the eukaryotic energy-sensing machinery") to consistently emphasize the shared evolutionary origin of these proteins while avoiding language implying that we have demonstrated a conserved energy-sensing mechanism. Several other passages have also been modified. We believe these revisions more accurately reflect the scope of our findings.

Having said that, I find this work outstanding and complete, and cannot support its publication more strongly - it is not every day that one discovers a key ribosomal partner that helps cells survive prolonged periods of metabolic inactivity. I foresee that this work will be one of the cornerstones of our understanding of how archaeal cells preserve their most metabolically expensive macromolecular assemblies during periods of persistent stress.

Referee #2:

Souza et al. use cryo-EM analysis of archaeal cell lysates grown to stationary phase to examine ribosomes. They identify AHA, a ribosome-associated factor that binds across the ribosomal subunits, occludes the mRNA channel and tRNA-binding sites, and functions as a ribosome hibernation factor. AHA is composed of two modules: a C-terminal domain homologous to the bacterial hibernation-promoting factor (HPF), and an N-terminal CBS tetrad domain related to the eukaryotic energy sensor AMPKγ, which is bound to two AMP molecules in the cryo-EM maps. Phylogenetic analysis shows broad conservation of AHA-related proteins across archaea and suggests evolutionary links between the C-terminal HPF-like domain and bacterial hibernation factors, as well as between the CBS domain and eukaryotic energy-sensing proteins. The authors also show that AHA is required for cell survival during stationary phase and for efficient recovery upon nutrient restoration, and that loss of AHA alters the stationary-phase proteome. The observation that AMP binds within conserved pockets of the AHA CBS domain further suggests a mechanism by which ribosome hibernation may be coupled to cellular energy status.

This is an interesting study that provides important structural and functional insight into archaeal ribosome regulation. It also proposes a new connection between translational control and energy sensing across domains of life. There are several points however that the authors should address prior to publication.

We thank Referee #2 for the careful evaluation of our manuscript and for the constructive suggestions. We appreciate the reviewer's positive assessment of the structural, functional, and evolutionary aspects of the study, and have carefully addressed each point below.

Specific comments:

1. Did the authors attempt polysome gradient profiling of wild-type and ΔAHA cells across different growth stages, such as exponential phase, transition into stationary phase, stationary phase, and recovery? This experiment would help establish whether loss of AHA alters ribosome populations or translational states.

To address this point, we performed sucrose-gradient profiling of wild-type and ΔAHA cells during exponential and stationary phase. These data are now included as Supplementary Figure 6. The profiles reveal a selective loss of 70S ribosomes relative to free 30S and 50S subunits in stationary-phase ΔAHA cells. We describe these findings in the Results (page 10) and Methods (page 22).

2. The MS data show depletion of more than 30 ribosomal proteins, predominantly from the large ribosomal subunit rather than the small subunit. This is interesting, especially given that some studies in eukaryotic systems have reported preferential degradation of the small subunit under starvation conditions. One possibility is that loss of AHA causes or exacerbates a ribosome assembly defect. Polysome profiling would be useful here as well, as it could reveal changes in 50S/30S/70S populations in ΔAHA cells compared with wild type.

We agree that an assembly defect is an interesting possibility. However, distinguishing defective ribosome assembly from accumulation of ribosome-degradation intermediates would require pulse-labeling or pulse-chase experiments that directly track newly synthesized ribosomal components (for example, Jomaa et al., Nucleic Acids Research, 2014, doi: 10.1093/nar/gkt1295; Davis et al., Cell, 2016, doi: 10.1016/j.cell.2016.11.020). We believe that such experiments are beyond the scope of the present study.

3. Related to the MS analysis, it would be informative to test whether ribosomal RNA levels on agarose gels for example, particularly the large-subunit rRNA, are similarly affected in ΔAHA cells. This could help assess if AHA protect the ribosomal RNA from degradation under stress conditions.

As noted in response to comment 1, the new sucrose-gradient profiles show reduced ribosome-associated material in stationary-phase ΔAHA cells, including a pronounced loss of the 70S peak. This finding is consistent with a role for AHA in preserving ribosome integrity (including rRNA) under stress, although it does not establish that AHA ***directly*** protects rRNA from degradation. We consider it more likely that, in the absence of AHA, dissociation of ribosomal proteins followed by their proteolysis exposes rRNA to cellular nucleases. Distinguishing whether AHA inhibits rRNA degradation directly from intact 70S particles or instead stabilizes ribosomal proteins on the particle and thereby slows decay of deprotonated rRNA will require future work.

4. The cryo-EM analysis was performed using lysates from cells cultured in semi-defined media until stationary phase, and the data suggest that AHA responds to low-energy states. However, because translation is directly dependent on GTP hydrolysis, it would be useful for the authors to discuss why archaea may use an ATP/AMP-sensing factor to regulate ribosome hibernation. For comparison, bacterial translational shutdown is often linked to alarmone-based stress signaling. *This point could be discussed more explicitly.*

The reviewer raises an important point regarding the distinction between GTP-dependent translation and ATP/AMP-dependent regulation of ribosome hibernation, which deserved further clarification. We have therefore expanded the Discussion to emphasize that AHA is proposed (but not directly demonstrated) to sense the energetic requirements of translation itself, but rather the overall cellular energy state through adenine nucleotide binding. Unlike bacteria, where ribosome hibernation is primarily coordinated through the stringent response and (p)ppGpp signaling, no analogous alarmone system has been identified in archaea. We therefore propose that adenine nucleotides provide an alternative regulatory signal, allowing AHA to couple ribosome hibernation to cellular energy availability. We have added this discussion to the revised manuscript [Discussion, page 16-17, "An important distinction, however, emerges when considering how AHA activity may be regulated. In bacteria, the induction of ribosome hibernation is tightly coupled to transcriptional control of the abundance of hibernation factors through the stringent response and the alarmone (p)ppGpp (Eymann *et al.*, 2002; Izutsu *et al.*, 2001a; Nagarajan *et al.*, 2025). This regulatory system is absent in archaea (van der Does *et al.*, 2023)" and lines 491-505: "This observation raises the possibility that, although translation itself is driven by GTP hydrolysis, the AHA·ribosome interaction may be regulated by adenine nucleotide binding, allowing ribosome hibernation to respond to the overall energetic state of the cell. Indeed, the relative levels of ATP, ADP, and AMP are canonical readouts of cellular energy status (Steinberg & Hardie, 2023), and

translational downregulation is expected to be advantageous under low-energy conditions. We therefore hypothesize that adenine nucleotide binding modulates the affinity of AHA for the ribosome …"].

5. Because multiple nutrients may become limiting during stationary phase, did the authors examine defined nutrient-depletion conditions to determine which specific limitations affect AHA levels or ribosome association? This would help clarify whether AHA responds broadly to stationary phase or to particular metabolic or nutrient cues.

We have not assessed the role of individual nutrient limitations. Give the now reduced scope of claims regarding AHA's- energy sensing role, we defer such studies to future work.

6. None of the figures show clear fits of the AHA atomic model into the corresponding cryo-EM density in either the main or supplementary figures. The exception is Figure S2, which shows density for only approximately seven residues. *The authors should consider including additional model-to-density views to better support assignment and modeling of this factor. The structural close-ups as well are somewhat busy and could benefit from simplification.*

To better support the assignment and modeling of AHA, we have expanded Supplementary Figure 2 to now include a model-to-density view of the AHA loop that inserts into the peptidyl transferase center. This region is central to our structural interpretation and demonstrates excellent agreement between the atomic model and the cryo-EM density.

Referee #3:

This paper reports the discovery of a Ribosome hibernation factor in achaea that represents an evolutionary link between energy sensing in eukaryotes and ribosome hibernation in prokaryotes. I find this paper very interesting, technically well executed and suitable for the general audience of EMBO.

The authors discover this new protein using a particularly interesting strategy of using lysates of stationary phase cells. I found this quite exciting. I also find the evolutionary implications very interesting and impactful. Perhaps the only deficiency of this paper is a lack of in vitro characterization, but I think that's quite clearly for future work and not needed for this study.

We are grateful for the reviewer's constructive feedback, for the positive assessment of our work, and for recognizing both the novelty of our experimental approach and the significance of the evolutionary findings.

I am no expert no the cryo-electron microscopy and cannot comment on the rigor of these analyses. My expertise lies in phylogenetics and I have a few comments about the phylogenies in this paper. These are in general inferred carefully, but some of the interpretation needs clarification:

The first problem is that the authors infer a phylogeny of the HPF domain, in which they see a clear split between archaea and bacteria. They take this to indicate that this family originated in LUCA. While this pattern is suggestive, the authors have not ruled out a single, cross-domain horizontal transfer. *To do this, they would need to show that the branching order within each domain is approximately what we expect from our understanding of the tree of life. I could not verify this from figure 2H.*

We agree that the archaeal–bacterial separation alone does not formally exclude an early cross-domain horizontal gene transfer event. We have therefore expanded our phylogenetic analyses and revised the text accordingly. Specifically, we now include the complete HPF and CBS phylogenies as new Source_Data_Figure_2H_1 and Source_Data_Figure_5D, together with the corresponding protein sequences, software commands, and intermediate files generated during the analyses. A color-coded version of the HPF tree was also generated to facilitate visualization of taxonomic distributions (Source_Data_Figure_2H_2). These analyses show that, beyond the primary archaeal–bacterial split, HPF sequences display a relative taxonomic structure within each domain, with major archaeal and bacterial lineages predominantly occupying distinct regions of the tree.

At the same time, we do not observe complete congruence between the HPF phylogeny and the species tree. Several major taxonomic groups are split across multiple regions of the tree, indicating that the evolutionary history of HPF is more complex than strict vertical inheritance. Such incongruences may reflect the limited phylogenetic signal of a single protein domain, ancient duplication and differential loss, horizontal gene transfer, or lineage-specific diversification.

Accordingly, we have moderated our interpretation throughout the manuscript. Specifically, we revised the manuscript to present inheritance from LUCA as the most parsimonious evolutionary scenario supported by our data, rather than as the only possible explanation. Rather than presenting

the HPF phylogeny as definitive proof of a LUCA origin, we now state that the deep archaeal–bacterial separation is **consistent with** an ancient origin of HPF predating the divergence of Archaea and Bacteria, while acknowledging that alternative evolutionary scenarios cannot be completely excluded (Results, page 8: "the most parsimonious explanation is that HPF was inherited from the LUCA. However, alternative evolutionary scenarios, including an early cross-domain horizontal gene transfer event, cannot be formally excluded"; Discussion, page 15, "the most parsimonious explanation is that this factor originated in the LUCA. Although alternative evolutionary scenarios cannot be formally excluded, our phylogenetic analyses are most consistent with an ancient origin predating the divergence of Archaea and Bacteria"; and Discussion, page 17: "its C-terminal HPF-like domain exemplifies an ancient ribosome hibernation strategy that may predate the divergence of Archaea and Bacteria for stabilizing inactive ribosomes…").

In Figure 5 the authors then infer an unrooted tree of the CBS domain module of this new protein. Was puzzled by some of the descriptions in the text that went along with this phylogeny. For example, the authors state that AHA likely evolved from SriA. I don't understand this inference. No possible root of this tree would clearly place SriA inside AHA. Most roots would simply make them sister groups, with AHA closer to their shared node in terms of branch lnegths than SriA. Could the authors clarify their text? To be clear, this has no bearing on the impact of their paper.

The reviewer is correct and we agree that the phylogeny is unrooted and therefore does not support the inference that AHA evolved from within the SriA lineage. We have revised the text accordingly to state that AHA and SriA are closely related sister groups derived from a common ancestral CBS-tetrad lineage, without implying ancestor–descendant relationships (Results, page 13: "according to this tree, AHA and SriA likely share a common ancestral CBS-tetrad protein that subsequently fused to an HPF domain, giving rise to the dual-module hibernation factor described here").

Similarly, the authors also state that AMPK gamma evolved with the archaeal CBS3 family. The situation is exactly as for the two proteins mentioned above: Unless the root of the entire tree is within CBS3, Ampk gamma is a sister group to CBS3 and doesn't emerge from within it. Again, the authors need to clarify these sentences.

We appreciate the reviewer pointing out this ambiguity. We agree that, because the phylogeny is unrooted, it does not support inferring that the eukaryotic AMPKγ family emerged specifically from the archaeal A-CBS3 lineage. We have therefore revised the text to avoid implying an ancestor–descendant relationship. Specifically, we now describe AMPKγ and the archaeal A-CBS3 family as closely related lineages recovered within the same CBS-tetrad clade (Results, page 13: "Our CBS-tetrad phylogeny recovered eukaryotic AMPKγ in a clade together with an uncharacterized archaeal family (A-CBS3) and a limited number of bacterial representatives, indicating a close evolutionary relationship among these groups"), and interpret AHA, A-CBS3, and AMPKγ as members of a broader archaeal CBS-tetrad radiation, consistent with a shared evolutionary origin without implying the direction of evolutionary descent (Results, page 13: "the CBS tetrad of AHA, the archaeal A-CBS3 proteins, and the eukaryotic AMPKγ family can be interpreted as related members of a broader archaeal CBS-tetrad radiation, consistent with a shared evolutionary origin").

Once again, we sincerely thank the Editor and all three reviewers for their thoughtful comments and constructive suggestions. We believe that the revised manuscript has been substantially strengthened by their input, and we hope that it is now suitable for publication in *The EMBO Journal*.

Prof. Joseph H Davis
Massachusetts Institute of Technology
Biology
77 Massachusetts Ave.
Cambridge, MA 02139

31st Aug 2026

Re: EMBOJ-2026-124255R
A conserved archaeal protein with evolutionary links to bacterial ribosome hibernation and eukaryotic energy sensing

Dear Joey,

Thank you for submitting your revised manuscript on archaea AHA hibernation factor to The EMBO Journal. It has now been again seen by two of the original referees (see comments below), who were both fully satisfied with the revisions. We shall therefore be happy to accept the manuscript for publication, following incorporation of a few remaining editorial points as follows:

- Please remove all figures from the manuscript text file, and collate their legends at the end of the text (following the reference section). Each main figure should be uploaded as a separate image-only file in sufficient resolution and quality for production.

- All 'supplemental' material (Figures S1-S9 and Tables S1-S2) should be collated in a separate 'Appendix' PDF, which should be prefaced with a title page stating 'Appendix for [manuscript title]' and a table of content including page number for each item. Make sure to consistently name/reference the included items as 'Appendix Figure S1/2/...' and 'Appendix Table S1/2'.

- Please change 'Source Data Table 1' into an Expanded View Dataset - renaming it to 'Dataset EV1' both in the file and when referencing it in the text. In the XLSX file, please include an additional 'Legend' tab stating this name and giving a brief explanation of the contents/type of data included.

- Please adjust the order of the manuscript sections, and also make sure to use the correct section headers:
Single title page with complete author information, Abstract, Introduction, Results, Discussion, Methods, Data Availability, Acknowledgements, Disclosure and Competing Interests Statement, References, Main Figure Legends, Tables, Expanded Figure Legends.

- As we have switched from a free-text author contribution statement towards a more formal statement based on Contributor Role Taxonomy (CRediT) terms, please remove the present Author Contribution section and instead specify each author's contribution(s) directly in the Author Information page of our submission system during upload of the final manuscript. See https://casrai.org/credit/ for more information.

- Please carefully go through the reference list and make sure that all references have their complete citation information - including year, journal name & volume, and page/elocator numbers - such information is currently missing for several of them. Please

- For preprint citations: Please change in-text reference format according to "preprint: XXX et al, 2026"; and in the reference list, name of the platform plus DOI + preprint label - e.g. "bioRxiv doi: 1234/002.dfj123 [PREPRINT]". Please double-check whether any of the referenced preprints may have been published as peer-reviewed articles in the meantime.

- In the Data Availability section, please include general access URLs for each of the utilized databases (e.g. PDB, EMDB), followed by the respective accession codes for the various datasets. Also, please make sure that the deposited entries become publicly accessible at this stage.

- Please indicate the exact p-values in the legends for 3F.

- Thank you for having provided all required Source Data. However, please note that Source Data files need to be saved according to the scheme 'one figure/one folder' and then uploaded as .zip files. E.g. all the Source data files for figure 1 need to be saved in a single folder, which then needs to be zipped and uploaded as "SD figure 1.zip" file. Any Source Data for Appendix figures (if applicable) should be combined in one single "SD Appendix.zip" file.

- Finally, please provide suggestions for a short 'blurb' text prefacing and summing up the conceptual aspect of the study in two sentences (max. 250 characters), followed by 3-5 one-sentence 'bullet points' with brief factual statements of key results of the paper; they will form the basis of an editor-written 'Synopsis' accompanying the online version of the article. Please also upload a synopsis image, which can be used as a "visual title" for the synopsis section of your paper. The image should be in JPG

format and exactly 550 pixels wide and 300 pixels high, and please make sure that it remains clear enough even in these modest dimensions.

I am returning the manuscript to you for a final round of minor revision, hoping you will be able to re-upload the modified final files during the course of this week. Once we will have received them, we should be ready to swiftly proceed with formal acceptance and production of the manuscript.

With kind regards,

Hartmut

Hartmut Vodermaier, PhD
Senior Editor, The EMBO Journal
h.vodermaier@embojournal.org

*** PLEASE NOTE: All revised manuscript are subject to initial checks for completeness and adherence to our formatting guidelines. Revisions may be returned to the authors and delayed in their editorial re-evaluation if they fail to comply to the following requirements. As a first step please read our guidelines for revised submissions:
https://link.springer.com/journal/44318/submission-guidelines#cms-Revised-submissions

1) Every manuscript requires a Data Availability section (even if only stating that no deposited datasets are included). Primary datasets or computer code produced in the current study have to be deposited in appropriate public repositories prior to resubmission, and reviewer access details provided in case that public access is not yet allowed.

2) Each figure legend must specify
- size of the scale bars that are mandatory for all micrograph panels
- the statistical test used to generate error bars and P-values
- the type error bars (e.g., S.E.M., S.D.)
- the number (n) and nature (biological or technical replicate) of independent experiments underlying each data point
- Figures may not include error bars for experiments with n<3; scatter plots showing individual data points should be used instead.

3) Revised manuscript text (including main tables, and figure legends for main and EV figures) has to be submitted as editable text file (e.g., .docx format). We encourage highlighting of changes (e.g., via text color) for the referees' reference.

4) Each main and each Expanded View (EV) figure should be uploaded as individual production-quality files (preferably in .eps, .tif, .jpg formats). For suggestions on figure preparation/layout, please refer to our Figure Preparation Guidelines:
https://media.springernature.com/original/springer-cms/rest/v1/content/27825798/data/v1

5) Point-by-point response letters should include the original referee comments in full together with your detailed responses to them (and to specific editor requests if applicable), and also be uploaded as editable (e.g., .docx) text files.

6) Please complete our Author Checklist, and make sure that information entered into the checklist is also reflected in the manuscript; the checklist will be available to readers as part of the Review Process File.

7) All authors listed as (co-)corresponding need to deposit, in their respective author profiles in our submission system, a unique ORCiD identifier linked to their name. Please see our Guide to Authors for detailed instructions.

8) Please note that supplementary information at EMBO Press has been superseded by the 'Expanded View' for inclusion of additional figures, tables, movies or datasets; with up to five EV Figures being typeset and directly accessible in the HTML version of the article.

9) To facilitate reproducibilty and cross-laboratory adoption of methodologies, please structure the Materials & Methods section as outlined in our guide to authors, including a completed Reagents and Tools Table.

10) Digital image enhancement is acceptable practice, as long as it accurately represents the original data and conforms to community standards. If a figure has been subjected to significant electronic manipulation, this must be clearly noted in the figure legend and/or the 'Materials and Methods' section. The editors reserve the right to request original versions of figures and the original images that were used to assemble the figure. Finally, we generally encourage uploading of numerical as well as gel/blot image source data.

At EMBO Press we ask authors to provide source data for the main manuscript figures. You will receive a separate email with instructions for providing source data with your revised manuscript, including how to upload and organize the files.

In the interest of ensuring the conceptual advance provided by the work, we recommend submitting a revision within 3 months (29th Nov 2026). Please discuss the revision progress ahead of this time with the editor if you require more time to complete the revisions. Use the link below to submit your revision:

Link Not Available

Referee #2:

The authors have revised the manuscript and adequately addressed all of my questions. This is an important study linking energy expenditure to translational shutdown and provides significant mechanistic insight into ribosome hibernation and translational control. I recommend the manuscript for publication in its current form.

Referee #3:

I am satisfied and recommend publication. I wish to congratulate all authors on a very nice piece of work

EMBO Press Author Checklist

Corresponding Author Name: Joseph H. Davis	
Journal Submitted to: The EMBO Journal	
Manuscript Number: EMBOJ-2026-124255	

USEFUL LINKS FOR COMPLETING THIS FORM
The EMBO Journal - Author Guidelines
EMBO Reports - Author Guidelines
Molecular Systems Biology - Author Guidelines
EMBO Molecular Medicine - Author Guidelines

Reporting Checklist for Life Science Articles (updated January
This checklist is adapted from Materials Design Analysis Reporting (MDAR) Checklist for Authors. MDAR establishes a minimum set of requirements in transparent reporting in the life sciences (see Statement of Task: 10.31222/osf.io/9sm4x). Please follow the journal's guidelines in preparing your
Please note that a copy of this checklist will be published alongside your article.

Abridged guidelines for figures
1. Data
The data shown in figures should satisfy the following conditions:
- the data were obtained and processed according to the field's best practice and are presented to reflect the results of the experiments in an accurate and unbiased manner.
- ideally, figure panels should include only measurements that are directly comparable to each other and obtained with the same assay.
- plots include clearly labeled error bars for independent experiments and sample sizes. Unless justified, error bars should not be shown for technical
- if n<5, the individual data points from each experiment should be plotted. Any statistical test employed should be justified.
- Source Data should be included to report the data underlying figures according to the guidelines set out in the authorship guidelines on Data

2. Captions
Each figure caption should contain the following information, for each panel where they are relevant:
- a specification of the experimental system investigated (eg cell line, species name).
- the assay(s) and method(s) used to carry out the reported observations and measurements.
- an explicit mention of the biological and chemical entity(ies) that are being measured.
- an explicit mention of the biological and chemical entity(ies) that are altered/varied/perturbed in a controlled manner.
- the exact sample size (n) for each experimental group/condition, given as a number, not a range;
- a description of the sample collection allowing the reader to understand whether the samples represent technical or biological replicates (including how many animals, litters, cultures, etc.).
- a statement of how many times the experiment shown was independently replicated in the laboratory.
- definitions of statistical methods and measures:
  - common tests, such as t-test (please specify whether paired vs. unpaired), simple χ2 tests, Wilcoxon and Mann-Whitney tests, can be unambiguously identified by name only, but more complex techniques should be described in the methods section;
  - are tests one-sided or two-sided?
  - are there adjustments for multiple comparisons?
  - exact statistical test results, e.g., P values = x but not P values < x;
  - definition of 'center values' as median or average;
  - definition of error bars as s.d. or s.e.m.

Please complete ALL of the questions below.
Select "Not Applicable" only when the requested information is not relevant for your study.

Materials

Newly Created Materials	Information included in the manuscript?	In which section is the information available? (Reagents and Tools Table, Materials and Methods, Figures, Data Availability Section)
New materials and reagents need to be available; do any restrictions apply?	Yes	Data Availability Section; no restrictions apply

Antibodies	Information included in the manuscript?	In which section is the information available? (Reagents and Tools Table, Materials and Methods, Figures, Data Availability Section)
For **antibodies** provide the following information:<br>- Commercial antibodies: RRID (if possible) or supplier name, catalogue number and or/clone number<br>- Non-commercial: RRID or citation	Not Applicable	

DNA and RNA sequences	Information included in the manuscript?	In which section is the information available? (Reagents and Tools Table, Materials and Methods, Figures, Data Availability Section)
**Short novel DNA or RNA including primers, probes**: provide the sequences.	Yes	Reagents and Tools Table; Table S2

Cell materials	Information included in the manuscript?	In which section is the information available? (Reagents and Tools Table, Materials and Methods, Figures, Data Availability Section)
**Cell lines:** Provide species information, strain. Provide accession number in repository **OR** supplier name, catalog number, clone number, and/**OR** RRID.	Not Applicable	
**Primary cultures:** Provide species, strain, sex of origin, genetic modification status.	Not Applicable	
Report if the cell lines were recently **authenticated** (e.g., by STR profiling) and tested for mycoplasma contamination.	Not Applicable	

Experimental animals	Information included in the manuscript?	In which section is the information available? (Reagents and Tools Table, Materials and Methods, Figures, Data Availability Section)
**Laboratory animals or Model organisms:** Provide species, strain, sex, age, genetic modification status. Provide accession number in repository **OR** supplier name, catalog number, clone number, **OR** RRID.	Not Applicable	
**Animal observed in or captured from the field:** Provide species, sex, and age where possible.	Not Applicable	
Please detail **housing and husbandry conditions**.	Not Applicable	

Plants and microbes	Information included in the manuscript?	In which section is the information available? (Reagents and Tools Table, Materials and Methods, Figures, Data Availability Section)
**Plants:** provide species and strain, ecotype and cultivar where relevant, unique accession number if available, and source (including location for collected wild specimens).	Not Applicable	
**Microbes:** provide species and strain, unique accession number if available, and source.	Yes	Reagents and Tools Table; Table S2; Materials and Methods

Human research participants	Information included in the manuscript?	In which section is the information available? (Reagents and Tools Table, Materials and Methods, Figures, Data Availability Section)
If collected and within the bounds of privacy constraints report on age, sex and gender or ethnicity for all study participants.	Not Applicable	

Core facilities	Information included in the manuscript?	In which section is the information available? (Reagents and Tools Table, Materials and Methods, Figures, Data Availability Section)
If your work benefited from core facilities, was their service mentioned in the acknowledgments section?	Yes	Acknowledgements section

Design

Study protocol	Information included in the manuscript?	In which section is the information available? (Reagents and Tools Table, Materials and Methods, Figures, Data Availability Section)
If study protocol has been **pre-registered, provide DOI in the manuscript**. For clinical trials, provide the trial registration number **OR** cite DOI.	Not Applicable	
Report the **clinical trial registration number** (at ClinicalTrials.gov or equivalent), where applicable.	Not Applicable	

Laboratory protocol	Information included in the manuscript?	In which section is the information available? (Reagents and Tools Table, Materials and Methods, Figures, Data Availability Section)
Provide DOI OR other citation details if **external detailed step-by-step protocols** are available.	Yes	Material and Methods

Experimental study design and statistics	Information included in the manuscript?	In which section is the information available? (Reagents and Tools Table, Materials and Methods, Figures, Data Availability Section)
Include a statement about **sample size** estimate even if no statistical methods were used.	Yes	Materials and Methods, Statistics and reproducibility section
Were any steps taken to minimize the effects of subjective bias when allocating animals/samples to treatment (e.g. **randomization procedure**)? If yes, have they been described?	Yes	Materials and Methods, Statistics and reproducibility section
Include a statement about **blinding** even if no blinding was done.	Yes	Materials and Methods, Statistics and reproducibility section
Describe **inclusion/exclusion criteria** if samples or animals were excluded from the analysis. Were the criteria pre-established?<br><br>If sample or data points were omitted from analysis, report if this was due to attrition or intentional exclusion and provide justification.	Yes	Materials and Methods, Statistics and reproducibility section
For every figure, are **statistical tests** justified as appropriate? Do the data meet the assumptions of the tests (e.g., normal distribution)? Describe any methods used to assess it. Is there an estimate of variation within each group of data? Is the variance similar between the groups that are being statistically compared?	Yes	Materials and Methods, Statistics and reproducibility section; figure legends

Sample definition and in-laboratory replication	Information included in the manuscript?	In which section is the information available? (Reagents and Tools Table, Materials and Methods, Figures, Data Availability Section)
In the figure legends: state number of times the experiment was **replicated** in laboratory.	Yes	Figure legends
In the figure legends: define whether data describe **technical or biological replicates**.	Yes	Materials and Methods; figure legends

Ethics

Ethics	Information included in the manuscript?	In which section is the information available? (Reagents and Tools Table, Materials and Methods, Figures, Data Availability Section)
Studies involving **human participants**: State details of **authority granting ethics approval** (IRB or equivalent committee(s), provide reference number for approval.	Not Applicable	
Studies involving **human participants**: Include a statement confirming that **informed consent** was obtained from all subjects and that the experiments conformed to the principles set out in the WMA Declaration of Helsinki and the Department of Health and Human Services Belmont Report.	Not Applicable	
Studies involving **human participants:** For publication of **patient photos**, include a statement confirming that consent to publish was obtained.	Not Applicable	
Studies involving experimental **animals**: State details of **authority granting ethics approval** (IRB or equivalent committee(s), provide reference number for approval. Include a statement of compliance with ethical regulations.	Not Applicable	
Studies involving **specimen and field samples:** State if relevant **permits** obtained, provide details of authority approving study; if none were required, explain why.	Not Applicable	

Dual Use Research of Concern (DURC)	Information included in the manuscript?	In which section is the information available? (Reagents and Tools Table, Materials and Methods, Figures, Data Availability Section)
Could your study fall under dual use research restrictions? Please check biosecurity documents and list of **select agents and toxins** (CDC): https://www.selectagents.gov/sat/list.htm	Not Applicable	
If you used a select agent, is the security level of the lab appropriate and reported in the manuscript?	Not Applicable	
If a study is subject to dual use research of concern regulations, is the name of the **authority granting approval and reference number** for the regulatory approval provided in the manuscript?	Not Applicable	

Reporting

The MDAR framework recommends adoption of discipline-specific guidelines, established and endorsed through community initiatives. Journals have their own policy about requiring specific guidelines and recommendations to complement MDAR.

Adherence to community standards	Information included in the manuscript?	In which section is the information available? (Reagents and Tools Table, Materials and Methods, Figures, Data Availability Section)
State if relevant guidelines or checklists (e.g., **ICMJE, MIBBI, ARRIVE, PRISMA**) have been followed or provided.	Not Applicable	
For **tumor marker prognostic studies,** we recommend that you follow the **REMARK** reporting guidelines (see link list at top right). See author guidelines, under 'Reporting Guidelines'. Please confirm you have followed these guidelines.	Not Applicable	
For **phase II and III randomized controlled trials**, please refer to the **CONSORT** flow diagram (see link list at top right) and submit the CONSORT checklist (see link list at top right) with your submission. See author guidelines, under 'Reporting Guidelines'. Please confirm you have submitted this list.	Not Applicable	

Data Availability

Data availability	Information included in the manuscript?	In which section is the information available? (Reagents and Tools Table, Materials and Methods, Figures, Data Availability Section)
Have **primary datasets** been deposited according to the journal's guidelines (see 'Data Deposition' section) and the respective accession numbers provided in the Data Availability Section?	Yes	Data Availability Section
Were **human clinical and genomic datasets** deposited in a public access-controlled repository in accordance to ethical obligations to the patients and to the applicable consent agreement?	Not Applicable	
Are **computational models** that are central and integral to a study available without restrictions in a machine-readable form? Were the relevant accession numbers or links provided?	Not Applicable	
If publicly available data were reused, provide the respective **data citations in the reference list**.	Yes	Materials and Methods; figure legends; reference list
