# Supplementary material for: A conserved archaeal protein with evolutionary links to bacterial ribosome hibernation and eukaryotic energy sensing
Source: EMBO J. 2026 Oct 2;45(21):7564–81. doi: 10.1038/s44318-026-00918-6 (PMC13728122; doi:10.1038/s44318-026-00918-6)

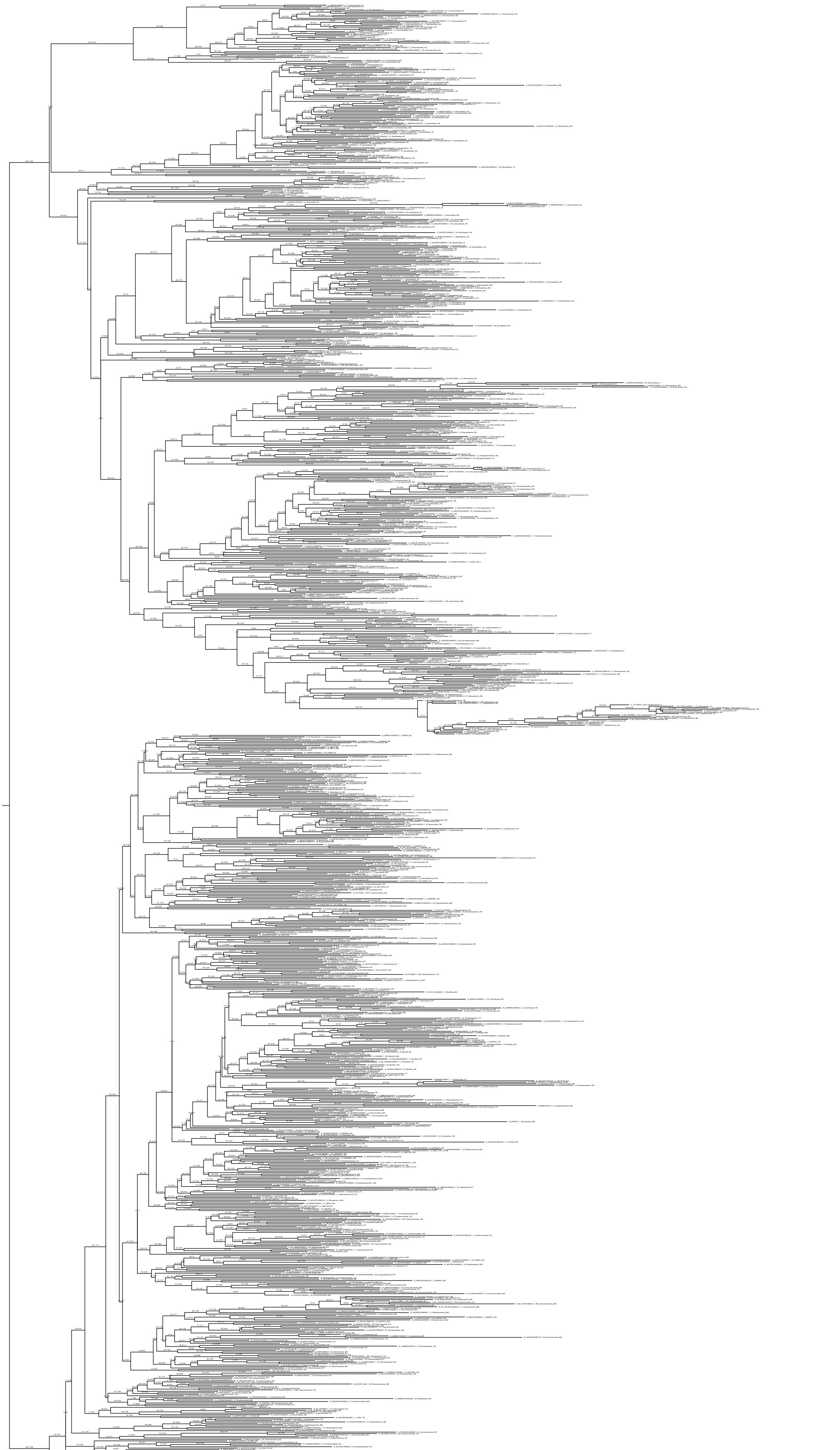

Supplement: Supplementary file 3 [file 44318_2026_918_MOESM4_ESM.zip › Figure2/Figure2H/Source_Data_Figure2H_1.pdf]
