# Supplementary material for: A conserved archaeal protein with evolutionary links to bacterial ribosome hibernation and eukaryotic energy sensing
Source: EMBO J. 2026 Oct 2;45(21):7564–81. doi: 10.1038/s44318-026-00918-6 (PMC13728122; doi:10.1038/s44318-026-00918-6)
Supplement: Supplementary file 5 [file 44318_2026_918_MOESM6_ESM.zip › Figure5/Figure5D/Source_Data_Figure_5D.pdf]

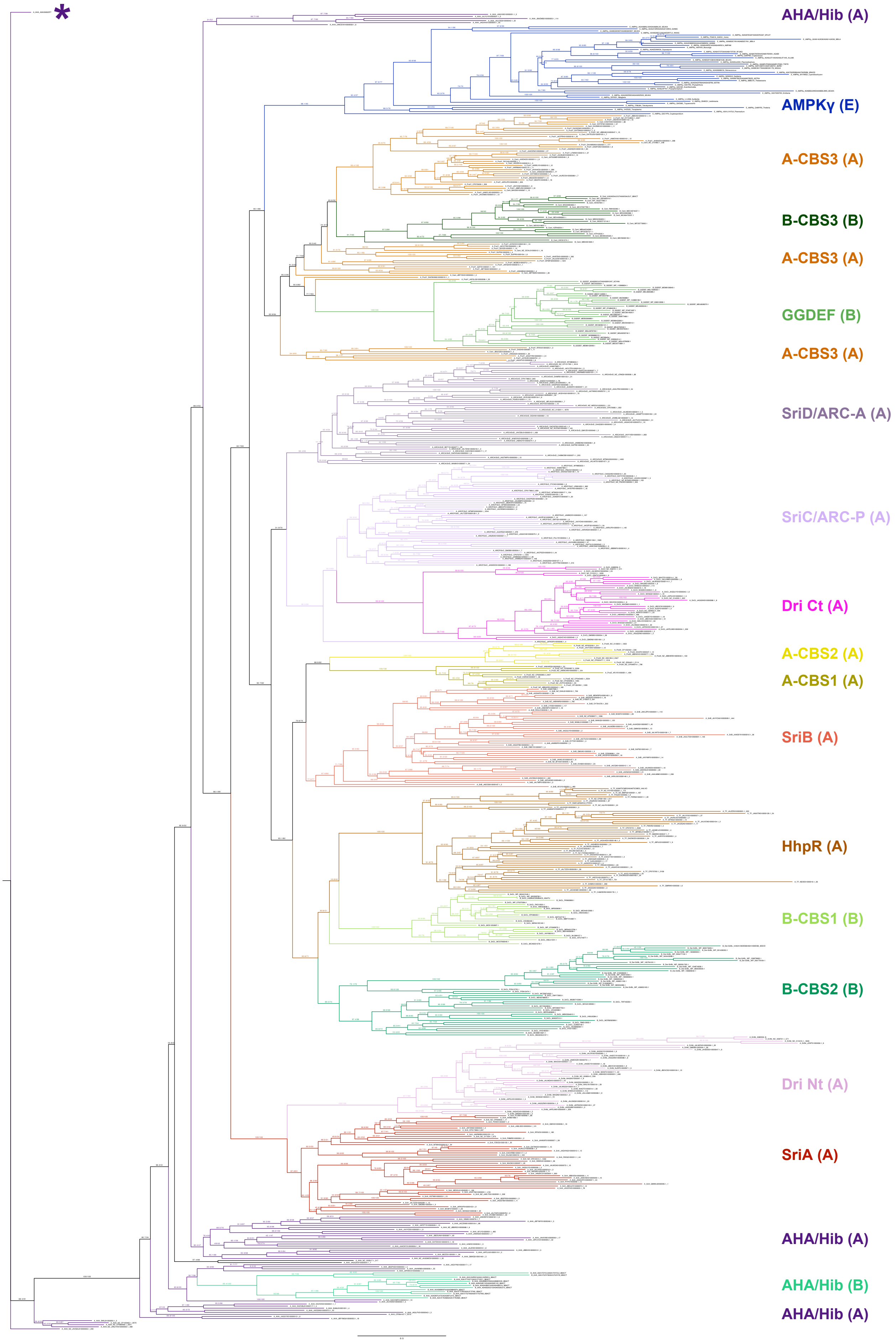
*
AHA/Hib (A)
AMPKγ (E)
A-CBS3 (A)
B-CBS3 (B)
A-CBS3 (A)
GGDEF (B)
A-CBS3 (A)
SriD/ARC-A (A)
SriC/ARC-P (A)
Dri Ct (A)
A-CBS2 (A)
A-CBS1 (A)
SriB (A)
HhpR (A)
B-CBS1 (B)
B-CBS2 (B)
Dri Nt (A)
SriA (A)
AHA/Hib (A)
AHA/Hib (B)
AHA/Hib (A)
